# Commodity Asian option pricing and simulation in a 4-factor model with jump clusters

**DOI:** 10.1007/s10479-022-05152-x

**Published:** 2023-01-07

**Authors:** Riccardo Brignone, Luca Gonzato, Carlo Sgarra

**Affiliations:** 1 Department of Quantitative Finance, Institute for Economic Research, University of Freiburg, Rempartstr. 16, 79098 Freiburg im Breisgau, Germany; 2 Department of Statistics and Operations Research, University of Vienna, Kolingasse 14-16, 1090 Vienna, Austria; 3 Department of Mathematics, Politecnico di Milano, Piazza Leonardo da Vinci, 32, 20133 Milan, Italy

**Keywords:** Commodity derivatives, Multifactor affine stochastic volatility models, Self-exciting jumps, Simulation, Asian options, C15, C63, G13, Q02

## Abstract

Mean reversion, stochastic volatility, convenience yield and presence of jump clustering are well documented salient features of commodity markets, where Asian options are very popular. We propose a model which takes into account all these stylized features. We first state our model under the historical measure, then, after introducing a structure preserving change of measure, we provide a risk-neutral version of the same model and we show how to price geometric and arithmetic Asian options. To this end, we derive semi-closed formulas for the geometric Asian options price and develop a computationally efficient simulation scheme for the price process, allowing to price the arithmetic counterparts using control variate technique. Finally, we propose a simple econometric experiment to document presence of jump clusters in commodity prices and evaluate the performances of the proposed simulation scheme on some parameter sets calibrated on real data.

## Introduction

**Fig. 1:**
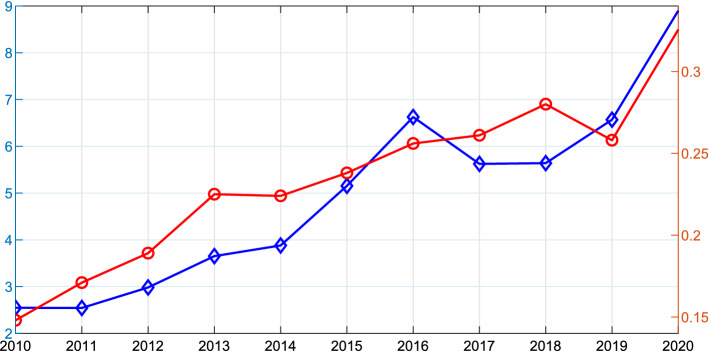
Historical time series of volumes of commodity futures (blue line) and options (red line) in billions of contracts. Source: World Federation of Exchanges (2019) for data until 2019 and https://focus.world-exchanges.org/articles/commodity-derivatives for data on 2020

Commodity derivatives markets had a tremendous growth in recent years, both in trading volume and variety of offered products, raising the need for models able to replicate correctly observed market prices. To quantify this growth, we present in Fig. 1 the time series of trading volumes of commodity futures and options. The total number of trades has more than doubled over the last ten years. Moreover, we note that in 2020 volumes increased substantially with respect to 2019 (by 35.7% for futures and 26.2% for options). This is possibly due to the fact that during highly uncertain times (like the COVID-19 outbreak) investors are more likely to hedge risk than in calm periods (see Gonzato and Sgarra, 2021 and references therein).

Much literature has been devoted to the study of empirical properties of commodity prices. Bessembinder et al. (1995) find clear evidence of mean reversion across many commodity markets. This is also confirmed by other prominent studies such as Schwartz (1997), Casassus and Collin-Dufresne (2005), and others. In commodity markets mean reversion is mainly induced by convenience yields, which stem from both the reduction in cost of acquiring inventory and the value of being able to profit from temporary local shortage of the commodity (Yan, 2002). Lutz (2010) surveys different methodologies proposed in literature to jointly model mean reversion and convenience yield and concludes that the so-called "autonomous convenience yield" approach provides good empirical performances for a wide variety of commodities. Based on this finding, we model mean reversion in the spot price directly by means of an Ornstein-Uhlenbeck process and assume that the convenience yield follows a mean reverting process which is independent of the spot price. Since typically convenience yield may assume both positive and negative values, following Casassus and Collin-Dufresne (2005), we assume a Gaussian Ornstein-Uhlenbeck process. Another salient feature of commodity markets is stochastic volatility of the log-returns, as documented, among others, by Trolle and Schwartz (2009) and Cortazar et al. (2017). Finally, there is intuition for the presence of self-excitation in the jumps process. This is the phenomenon, also named "jump clustering", in which whenever a jump in the asset price occurs, the possibility of observing subsequent jumps increases. Filimonov et al. (2014) found clear evidence of this effect in many commodity markets and concluded that: "At least 60-70 per cent of commodity price changes are now due to self-generated activities rather than novel information". Additional studies confirm the importance of including self-excitation in commodity price modeling for derivatives pricing, hedging and forecasting (Jiao et al., 2019 and Gonzato and Sgarra 2021). In the course of this paper we provide further evidence of jump clustering effects in oil and precious metal markets. Following standard asset pricing literature (see Fulop and Li, 2019 and the references therein), we model the stochastic jump intensity through a Hawkes process with exponential kernel.

The resulting proposed model is a 4-factor affine model for commodity prices. The model takes into account all aforementioned stylized features and its primary scope is to accurately price commodity derivatives. To this end, after describing our model under the historical measure, we propose a structure preserving change of measure which allows formulation of the model under a risk–neutral pricing measure. The necessity of using general multi–factor models for pricing commodity derivatives is highlighted in Cortazar et al. (2017) and Schöne and Spinler (2017). The model can be considered as an extension of that proposed in Casassus and Collin-Dufresne (2005) with stochastic volatility and self-exciting jumps. Due to its affine structure we obtain closed formulas for the futures price and semi-closed formulas for European options on futures, useful for model calibration purposes, as detailed in Sect. 5. Moreover, as we discuss later on, the process can be accurately simulated and allows for efficient evaluation procedures for Asian options. This is very important for several reasons: (i) in commodity markets Asian options are traded much more frequently than in equity or interest rate markets; (ii) model sophistication typically precludes exact formulas for the price of exotic derivatives and pricing must be performed through simulation; (iii) general multi-factor models present multiple sources of randomness leading to a large variance in the simulation step and, if the simulation is not accurate enough, to relevant mispricing of the derivative instrument (as we document in the case of the Euler scheme).

Asian options are very popular among commodity derivatives traders and risk managers,Here we don’t mean that Asian options are the most traded options in general, but that they are strongly linked to the commodity market and they are quite popular among practitioners, especially in OTC markets (see e.g. Kaminski, 1999). as their payoff depends on the arithmetic average of the prices assumed by the underlying during the life of the contract and represent a cheaper alternative to European options. The averaging process is responsible for their popularity: it smooths possible market manipulations occurring near the expiry date, reduces payoff’s volatility and allows for better cash flow matching. Hence, many institutions started quoting average price options for highly volatile assets such as commodities (we refer to Roncoroni et al., 2015, Chapter 18 for a nice description of the traded products). However, Asian options are traded mainly in Over the Counter (OTC) markets (e.g. Kaminski, 1999).

Even if exact computation of the price of an arithmetic Asian option is precluded, we obtain semi-closed formulas for the price of a discretely monitored geometric Asian option under the proposed model. In this context, we contribute to the literature deriving the moment generating function of the arithmetic average of the asset returns, extending the results in Fusai and Kyriakou (2016) to the case of multi-factor affine models with mean reversion. Then, for accurate model simulation, we rely on two different streams of literature on the exact simulation of option pricing models with stochastic volatility (Broadie and Kaya, 2006) and on the exact simulation of affine point processes (Dassios and Zhao, 2013). We use their results as building block for the construction of an almost exact simulation scheme for the proposed model which allows to simulate asset price trajectories observed at discrete times and produce almost unbiased estimates for exotic derivatives prices. Unfortunately, exact simulation is possible only in absence of mean reversion, in the opposite case we propose a simple approximation, whose accuracy is investigated through extensive numerical experiments. Due to the approximation, accuracy increases with the number of time discretization steps. Since we consider four stochastic factors we observe high Monte Carlo variance in the simulation step. Therefore, we employ the previously computed geometric Asian option price as control variate to reduce the variance of the estimator for the arithmetic Asian option price (Kemna and Vorst, 1990). Effectiveness of the proposed pricing methodologies is confirmed through extensive numerical experiments using realistic model parameters calibrated on real market quotes. We show that the bias of the proposed simulation scheme decays faster than that of the Euler scheme (used as benchmark) with a higher convergence rate of the Root Mean Squared Error (RMSE) and better overall performances in terms of trade-off between accuracy and computational effort.

The rest of the paper is organized as follows. In Sect. 2 we introduce the model specification and derive pricing formulas for European call options on futures (which will be later used for model calibration). In Sect. 3 we present an efficient simulation algorithm for the proposed model, while Sect. 4 deals with Asian option pricing. In Sect. 5 we perform numerical studies, where we document the presence of jump clusters and investigate the performances of the proposed simulation scheme. Section 6 concludes.

## Model setup

In this section we outline our model under the historical probability measure. Then, we introduce a structure preserving change of measure and describe our model under the risk–neutral measure. Finally, we show how to price futures and options on futures contracts under this model specification.

### Model dynamics under the historical measure

Let $(\Omega , {\mathcal {F}}, ({\mathcal {F}}_t)_{t\in [0, T]}, {\mathbb {P}})$ be a filtered probability space, which supports all the processes we encounter in the sequel. If we denote by $S_t$ the spot price process, the log-return process is $X_t := \log (S_t/S_0)$. Under the historical measure ${\mathbb {P}}$ the dynamics of the log-returns is defined by the following system of stochastic differential equations:

1 $$\begin{aligned} dX_t&= \left( \mu - \frac{V_t}{2} - \lambda _t \mu ^{*}- \delta _t - \alpha X_t \right) dt + \sqrt{V_t}dW^{x}_t + J_x dN_t, \end{aligned}$$

2 $$\begin{aligned} dV_t&= k_v (\theta _v-V_t)dt + \sigma _v\sqrt{V_t}dW^{v}_t,\end{aligned}$$

3 $$\begin{aligned} d \delta _t&= k_{\delta }(\theta _{\delta } - \delta _t)dt + \sigma _{\delta } dW^{\delta }_t \end{aligned}$$

4 $$\begin{aligned} d\lambda _t&= k_{\lambda }(\theta _{\lambda }-\lambda _t)dt + \beta dN_t, \end{aligned}$$

where $J_x N_t$ is a marked point process with stochastic intensity $\lambda _t$ and random jump size $J_x$ and $\mu ^{*} = e^{\mu _J + \sigma ^2_J/2}-1$ its compensator. We assume a set of initial conditions satisfied by each one of the processes described by the SDE system: $\{X_0, V_0, \delta _0, \lambda _0\}$. Following Casassus and Collin-Dufresne (2005, Proposition 2), we assume that the convenience yield $\delta _t$ evolves according to an independent Gaussian Ornstein–Uhlenbeck process (which can assume both positive and negative values). The parameter $\alpha \ge 0$ controls the speed of mean reversion. Following Larsson and Nossman (2011), Brooks and Prokopczuk (2013), Gonzato and Sgarra (2021), we assume a Cox et al. (1985) (CIR) square-root diffusion process for the variance $V_t$, which is correlated with the price process through the coefficient $\rho \in [-1,1]$, i.e. $\textrm{E}\left[ dW_t^{x} dW_t^{v}\right] =\rho dt$. We model abrupt changes in log-returns $X_t$ by including an independent compound Poisson process with stochastic intensity $\lambda _t$, such that the counting process exhibits a self-exciting behavior, which means that a jump increases the probability of observing subsequent jumps. Although the jump intensity $\lambda _t$ is stochastic and path-dependent, it is possible to prove that the vector process $\{X_t , V_t , \delta _t , \lambda _t\}$ is Markovian and affine (as we discuss later on). The characterization of price jumps is completed by specifying a probability density function for their sizes, which are i.i.d. Gaussian and denoted by $J_x\sim {\mathcal {N}}(\mu _J, \sigma _J^2)$. The coefficient $\mu$ describes a constant drift with respect to the historical measure. We further assume that the following non-explosion condition for the Hawkes process $N_t$ in (4):

5 $$\begin{aligned} k_{\lambda }>\beta , \end{aligned}$$

and the well-known Feller condition on the parameters of (2) (granting the strict positivity of $V_t$ for all $t>0$) $2 k_v \theta _v \ge \sigma ^2_v$ hold.

The model nests many other models proposed in literature to describe a large variety of commodities dynamics, we mention just a few of them. The classical Gibson and Schwartz (1990) model is obtained with $V_t=\lambda _t=0$ and constant interest rates, the model in Casassus and Collin-Dufresne (2005), well suited for a large number of commodities such as oil, copper, gold, silver is obtained imposing $V_t=\lambda _t=0$. Eydeland and Geman (1998) propose a model for gas and electricity which is obtained with $\delta _t=\lambda _t=0$, while the one of Geman (2000) (for oil) with $\lambda _t=0$. Moreover, we mention the model proposed in Larsson and Nossman (2011) for oil prices which is a special case with $\delta _t=\alpha =0$ and constant $\lambda _t$.

Before performing our analysis on the proposed model we want to examine the issue related to existence and uniqueness for the system of stochastic differential equations characterizing our model. We have the following

#### Proposition 1

There exists a solution to the system of (1–4) when a non explosion condition holds and this solution is adapted to the filtration generated by the driving processes. This solution is unique when standard initial data (${\mathcal {F}}_0$-measurable initial data) are assigned.

#### Proof

See Appendix A. $$\square $$

As far as positivity of (2) and (4) is concerned (neither positivity of $X_t$ nor positivity of $\delta _t$ are required at all), the classical Feller condition on the parameters describing the volatility dynamics $2 k_v \theta _v > \sigma ^2_v$ will grant the strict positivity of $V_t$ for $t>0$, while (4), as an Ornstein-Uhlenbeck SDE driven by a jump process with positive long-term mean $\theta _{\lambda }$ and with only positive jumps in the driver, cannot exhibit a negative solution.

### Risk–neutral dynamics

In order to deal with derivatives pricing we need to introduce a risk-neutral measure. To this end, we extend the results in Zhang et al. (2009) and Hainaut and Moraux (2018) by introducing an Esscher–type measure change. This is defined as follows. If we denote by $L_t := J_x dN_t$ the jump term in (1), and by $\psi ^{ {\mathbb {P}}} (z) := \textrm{E}[ e^{ z J_x } ]$ the moment generating function of the jump size density, we can define the following family of exponential martingales:

$$\begin{aligned}&M_t (\xi , \phi _x , \phi _{\delta } , \phi _v ) \\&\quad := \exp {\left[ \kappa _1 (\xi ) \lambda _t + \xi L_t + \kappa _2 (\xi ) t - \frac{1}{2} \int _0^t \phi ^{2}_x (u) du - \int _0^t \phi _x (u) dW^{x}_{u} \right] } \\&\qquad \times \exp {\left[ - \frac{1}{2} \int _0^t \phi ^{2}_v (u) du - \int _0^t \phi _v (u) dW^{v}_{u} - \frac{1}{2} \int _0^t \phi ^{2}_{\delta } (u) du - \int _0^t \phi _{\delta } dW^{\delta }_{u} \right] } \end{aligned}$$

where $\kappa _1 (\xi ) , \kappa _2 (\xi ), \eta$ (with $\kappa _1 (\xi ) , \kappa _2 (\xi )$ functions of $\xi$) denote the risk premium related to the jumps and $\phi _x , \phi _v , \phi _{\delta }$ (stochastic processes adapted to the reference filtration ${\mathcal {F}}_t$) denote the risk premium related to the diffusion components of log-returns, volatility and convenience yield respectively. We state the following result.

#### Proposition 2

If, for any $\xi$, there exist functions $\kappa _1 (\xi ) , \kappa _2 (\xi )$, which are solutions of the following system of algebraic equations:

6 $$\begin{aligned} {\left\{ \begin{array}{ll} &{}\kappa _1 k_{\lambda } - [\exp {(\kappa _1 (\xi ) \beta )} \psi (\xi ) -1 ] = 0 \\ &{}\kappa _2 + \kappa _1 k_{\lambda } \theta _{\lambda } = 0 , \end{array}\right. } \end{aligned}$$

then $M_t (\xi , \phi _x , \phi _{\delta } , \phi _v )$ is a local martingale. If, moreover, the non-explosion condition (5) holds for $N_t$ and the Novikov condition holds for $\phi _x , \phi _v , \phi _{\delta }$, $M_t$ is a true martingale.

#### Proof

See Appendix B $$\square $$

The following result shows that the measure change introduced by the likelihood process $\frac{d{\mathbb {Q}}}{d{\mathbb {P}}} \big |_{{\mathcal {F}}_t} = \frac{M_t (\xi , \phi _x , \phi _{\delta } , \phi _v )}{ M_0 (\xi , \phi _x , \phi _{\delta } , \phi _v )}$ preserves the model structure.

#### Proposition 3

The dynamics under the risk-neutral measure ${\mathbb {Q}}$ of $X^{{\mathbb {Q}}}_t , V^{{\mathbb {Q}}}_t , \lambda ^{{\mathbb {Q}}}_t , \delta ^{{\mathbb {Q}}}_t$ is given by the following system of stochastic differential equations:

7 $$\begin{aligned} dX^{{\mathbb {Q}}}_t&= \left( -\frac{1}{2}V^{{\mathbb {Q}}}_t - \mu ^{\star , {\mathbb {Q}} } \lambda ^{{\mathbb {Q}}}_t - \delta ^{{\mathbb {Q}}}_t - \alpha X^{{\mathbb {Q}}}_t\right) dt + \sqrt{V^{{\mathbb {Q}}}_t}dW^{x,{\mathbb {Q}}}_t + J_x^{{\mathbb {Q}}} dN^{{\mathbb {Q}}}_t , \end{aligned}$$

8 $$\begin{aligned} dV^{{\mathbb {Q}}}_t&=k^{{\mathbb {Q}}}_v(\theta ^{{\mathbb {Q}}}_v-V^{{\mathbb {Q}}}_t)dt + \sigma ^{{\mathbb {Q}}}_v\sqrt{V^{{\mathbb {Q}}}_t}dW^{v,{\mathbb {Q}}}_t,\end{aligned}$$

9 $$\begin{aligned} d \delta ^{{\mathbb {Q}}}_t&=k^{{\mathbb {Q}}}_{\delta }(\theta ^{{\mathbb {Q}}}_{\delta } - \delta ^{{\mathbb {Q}}}_t)dt + \sigma ^{{\mathbb {Q}}}_{\delta } dW^{\delta , {\mathbb {Q}}}_t , \end{aligned}$$

10 $$\begin{aligned} d\lambda ^{{\mathbb {Q}}}_t&=k^{{\mathbb {Q}}}_{\lambda } (\theta ^{{\mathbb {Q}}}_{\lambda }-\lambda ^{{\mathbb {Q}}}_t )dt + \beta ^{{\mathbb {Q}}} dN^{{\mathbb {Q}}}_t, \end{aligned}$$

where $J_x^{{\mathbb {Q}}} N^{{\mathbb {Q}}}_t$ denotes the jump process with respect to ${\mathbb {Q}}$ and $\mu ^{\star , {\mathbb {Q}} }= e^{\mu ^{{\mathbb {Q}}}_J + \frac{1}{2} \sigma _J^{{\mathbb {Q}},2} }-1$. The relations between the relevant parameters under ${\mathbb {P}}$ and ${\mathbb {Q}}$ are the following:

$$\begin{aligned}&dW^{x,{\mathbb {Q}}}_t = dW^{x,{\mathbb {P}}}_t + \phi _x (t) dt,\, dW^{v,{\mathbb {Q}}}_t = dW^{v,{\mathbb {P}}}_t + \phi _v (t) dt, \, dW^{\delta ,{\mathbb {Q}}}_t = dW^{\delta ,{\mathbb {P}}}_t + \phi _{\delta } (t) dt \\&\sigma _{\delta }^{{\mathbb {Q}}} = \sigma _{\delta },\, \sigma _v^{{\mathbb {Q}}} = \sigma _v,\, k^{{\mathbb {Q}}}_v = k_v + \phi _v \sigma _v,\, k^{{\mathbb {Q}}}_{\delta } = k_{\delta },\, k^{{\mathbb {Q}}}_{\lambda } = k_{\lambda },\, \rho ^{{\mathbb {Q}}} = \textrm{E}[dW^{x,{\mathbb {Q}}}_t , dW^{v,{\mathbb {Q}}}_t ]/dt= \rho \\&\theta _v^{{\mathbb {Q}}} = \theta _v \frac{k_v}{k_v + \phi _v \sigma _v},\, \theta _{\delta }^{{\mathbb {Q}}} = \theta _{\delta } + \frac{\phi _{\delta }}{\sigma _{\delta }},\, \theta _{\lambda }^{{\mathbb {Q}}} = \theta _{\lambda } e^{\kappa _1 (\xi ) \beta }\psi (\xi ),\, \beta ^{{\mathbb {Q}}} = e^{ \kappa _1 (\xi ) \beta } \psi (\xi ) \beta . \end{aligned}$$

Moreover, under ${\mathbb {Q}}$ the jump size $J_x^{{\mathbb {Q}}}$ is still normally distributed with mean $\mu ^{{\mathbb {Q}}}_J$ and volatility $\sigma _J^{{\mathbb {Q}}}$, with moment generating function given by $\psi ^{{\mathbb {Q}}} (z) = \psi (z + \xi ) /\psi (\xi )$.

#### Proof

See Appendix C. $$\square $$

#### Remark 1

We shall assume in the following that the risk premium terms $\phi _x , \phi _v$ are such that the following equality is satisfied for all $t \in [0,T]$:

$$\begin{aligned} \mu - \sqrt{V_t} [ \rho \phi _v (t) + \sqrt{ 1- \rho ^2} \phi _x (t)] + \lambda _t [\psi (1) - 1]= 0, \end{aligned}$$

in such a way that the dynamics of log-returns under ${\mathbb {Q}}$ can be written as in (7), by observing that $\psi (1) -1= \mu ^{*}$.

#### Remark 2

The last sentence in Proposition 3 implies that the relations between the mean and the variance of the jump size with respect to ${\mathbb {Q}}$ and ${\mathbb {P}}$ are the following: $\mu ^{\mathbb {Q}}_J = \mu _J + \xi \sigma _J^2$ and $\sigma ^{\mathbb {Q}}_J = \sigma _J$.

We point out that when $\alpha > 0$ the spot price does not satisfy the standard no-arbitrage condition and the spot price process is not a local martingale with respect to ${\mathbb {Q}}$. This is not a problem since commodities with storage costs of the good are not directly traded assets, therefore the drift of the log-returns can be of the mean-reverting type under the risk-neutral measure (see e.g. Schwartz, 1997; Lutz, 2010; Benth, 2011; Cai et al., 2014). As a result, the proposed framework is well suited also for option pricing, as witnessed also by the large amount of literature dealing with option pricing under mean reversion of the asset price (see Fusai et al., 2008; Wong and Lo, 2009; Chung and Wong, 2014; Brignone et al., 2021, among others). However, in the special case with $\alpha =0$ the price process is a local martingale under the risk-neutral measure ${\mathbb {Q}}$. We are also assuming that the short rate is equal to 0. This is in agreement with most theoretical models with stochastic convenience yields (e.g. Schwartz, 1997; Routledge et al., 2000).

#### Remark 3

The non-explosion condition under ${\mathbb {Q}}$ is now given by $k_{\lambda }^{{\mathbb {Q}}}>\beta ^{{\mathbb {Q}}}$, i.e. $k_{\lambda }^{{\mathbb {P}}} > \exp {(\kappa _1 (\xi ) \beta )} \beta ^{{\mathbb {P}}}$, that we’ll assume to be satisfied in the following.

In the next subsection, we show how to perform option pricing under the model (7)–(10). Despite the growing interest and success in modeling jump clustering in finance (see e.g. Fulop and Li, 2019 and the references therein), the application of such framework on commodity options is still unexplored and we provide a first contribution on this topic.

#### Remark 4

Since in the rest of the paper we shall work always under the risk-neutral dynamics, we shall drop the superscript ${{\mathbb {Q}}}$ from all the relevant quantities.

### Pricing options on futures contracts

We derive, next, the joint moment generating function of the quantities described in (7)–(10), we will make use of this result to price derivatives under the proposed model:

#### Proposition 4

Given a final date $T>t$ and the time to maturity $\tau =T-t$, the joint moment generating function of $(X_T, \delta _T, V_T, \lambda _T)$ is

11 $$\begin{aligned} \textrm{E}[e^{u_1 X_T + u_2 V_T + u_3 \delta _T + u_4 \lambda _T}|{\mathcal {F}}_t]&=\exp \Big ((u_1 + G(u_1, \tau ))X_t + A(u_1, u_2, u_3, u_4,\tau ) \nonumber \\&\quad + B(u_1, u_2,\tau )V_t + C(u_1, u_3,\tau )\delta _t + D(u_1, u_4, \tau )\lambda _t\Big ) \end{aligned}$$

where

$$\begin{aligned} {\left\{ \begin{array}{ll} \frac{\partial A(u_1, u_2, u_3, u_4,\tau )}{\partial \tau }= F_1(u_1, u_2, u_3, u_4, \tau ) ,\\ \frac{\partial B(u_1, u_2,\tau )}{\partial \tau } = F_2(u_1, u_2, \tau ),\\ \frac{\partial D(u_1, u_4,\tau )}{\partial t} = F_4(u_1, u_4, \tau ),\\ \end{array}\right. } \end{aligned}$$

and

$$\begin{aligned} \left\{ \begin{array}{l} F_1(u_1, u_2, u_3, u_4, \tau ) = k_v\theta _vB(u_1, u_2,\tau ) +k_{\delta } \theta _{\delta } C(u_1, u_3,\tau ) + k_{\lambda }\theta _{\lambda }D(u_1, u_4,\tau ) \\ \qquad \qquad \qquad \qquad \qquad \qquad + \frac{1}{2}\sigma _{\delta }^2 C(u_1, u_3, \tau )^2 ,\\ F_2(u_1, u_2, \tau ) = -\frac{1}{2}(u_1 + G(u_1,\tau )) + \frac{1}{2}(u_1 + G(u_1,\tau ))\\ \qquad \qquad \qquad \qquad \qquad \qquad (u_1 + G(u_1,\tau )) - k_vB(u_1, u_2,\tau )\\ \qquad \qquad \qquad \qquad \qquad \qquad + \frac{1}{2}\sigma _v^2 B(u_1, u_2,\tau )^2 + \rho \sigma _v B(u_1, u_2,\tau )u_1 \\ \qquad \qquad \qquad \qquad \qquad \qquad + \rho \sigma _vB(u_1, u_2, \tau ) G(u_1, \tau ),\\ F_4(u_1, u_4, \tau ) = -\mu ^{\star }(u_1 + G(u_1,\tau )) - k_{\lambda } D(u_1, u_4,\tau ) \\ \qquad \qquad \qquad \qquad \qquad \qquad + e^{\beta D(u_1, u_4,\tau )}(e^{(u_1 + G(u_1,\tau ))\mu _J + \sigma ^2_J/2(u_1+G(u_1,\tau ))^2} - 1),\\ \end{array}\right. \end{aligned}$$

with initial conditions $A(u_1, u_2, u_3, u_4,0) = 0$, $B(u_1, u_2,0) = u_2$, $D(u_1, u_4,0) = u_4$ and

$$\begin{aligned} G(u_1, \tau )&= u_1(e^{-\alpha \tau } - 1), \quad C(u_1, u_3,\tau ) = \frac{e^{-k_{\delta } \tau } \left( u_1 \left( -e^{\tau (k_{\delta }-\alpha )}\right) +u_3 (k_{\delta }-\alpha )+u_1\right) }{k_{\delta }-\alpha }. \end{aligned}$$

Moreover, in the case with $\alpha =0$, we have

$$\begin{aligned} B(u_1, u_2, \tau ) = \frac{\gamma \tan \left( \frac{1}{2} \tau \gamma -\tan ^{-1}\left( \frac{k_v-\sigma _v (\rho u_1+\sigma _v u_2)}{\gamma }\right) \right) +k_v-\rho \sigma _v u_1}{\sigma _v^2} \end{aligned}$$

where $$\gamma := \sqrt{-k_v^2+2 k_v \rho \sigma _v u_1+\sigma _v^2 u_1 \left( \rho ^2 (-u_1)+u_1-1\right) }$$.

#### Proof

See Appendix D. $$\square $$

#### Remark 5

*C* and *G* can be computed full explicitly and the solution is reported in Proposition 4. In addition, also *B* can be computed explicitly, however, when $\alpha >0$ the solution is in terms of hypergeometric functions. Hence, we find in practice more convenient to solve the corresponding Ordinary Differential Equation (ODE) numerically than through the analytic solution. *D* is discussed in the next remark.

#### Remark 6

The couple $(N_t, \lambda _t)$ is an affine Markov process (see e.g. Errais et al., 2010). Therefore, the model (7)–(10) is affine and it is possible to derive an expression for the moment generating function of log-returns as solution to a ODEs system. Nevertheless, an explicit solution for the ODE $F_4$ in Proposition 4 is not available and numerical solvers must be considered (see e.g. Errais et al., 2010; Da Fonseca and Zaatour, 2014).

In the following, we will exploit Proposition 4 to perform derivatives pricing. Consider $S_T = S_t e^{X_{T}}$ where $\tau = T - t$ is the time to maturity and $S_t$ is the price of an asset at time *t*. The price of futures contract *F*(*t*, *T*) is given by the standard relation:

$$\begin{aligned} F(t, T) = \textrm{E}[S_T | {\mathcal {F}}_t]. \end{aligned}$$

Under the proposed model, we have

12 $$\begin{aligned} \log F(t,T)&= \log S_t + \log \textrm{E}[e^{X_{T}} | {\mathcal {F}}_t] \nonumber \\&= \log S_t + (1+G(1,\tau ))X_t + A(1, 0, 0, 0, \tau ) +\nonumber \\&\quad + B(1,0,\tau ) V_t+ C(1, 0, \tau )\delta _t + D(1,0,\tau )\lambda _t \end{aligned}$$

where we compute the expectation by replacing $u_1 = 1$ and $u_2=u_3=u_4 = 0$ into (11). We turn now our attention to the pricing of European options. Consider a maturity *T* and a strike *K*, then the price will be given by

13 $$\begin{aligned} C_E = \textrm{E}[\max (0, S_T- K) | {\mathcal {F}}_t] = \int _{K}^{\infty } (S_t^x - K)f_{X_{T}}(x) dx \end{aligned}$$

where $f_{X_{T}}(x)$ is the probability density function of $X_{T}$ obtained by numerical inversion of the characteristic function of $X_T$, i.e. $\textrm{E}[e^{i u X_{T}}|{\mathcal {F}}_t]$, a special case of Proposition 4 with $u_1:=i u$ and $u_2 = u_3= u_4 = 0$. Given a (univariate) characteristic function we compute the corresponding probability density function through the Fourier-Cosine (COS) method proposed by Fang and Oosterlee (2008). Given the probability density function, the European option price can be obtained in several ways, for example by solving the remaining integral (e.g. 13) using the trapezium rule.

Consider now a European option on futures at the initial date *t*, with maturity of the option *T* and maturity of the underlying futures contract ${\tilde{T}}>T$, the price is given by:

14 $$\begin{aligned} C_{EF}&= \textrm{E}[\max (0, F(T, {\tilde{T}} ) - K)|{\mathcal {F}}_t]= \textrm{E}[\max (0, S_T \textrm{E}[e^{X_{{\tilde{T}} - T}}|{\mathcal {F}}_T] - K)|{\mathcal {F}}_t]\nonumber \\&= \textrm{E}[\max (0, S_t e^{Y_T} - K)|{\mathcal {F}}_t]=\int _{K}^{\infty } (S_t e^{y} - K)f_{Y_T}(y) dy \end{aligned}$$

where $$Y_T :=(1 + G(1,{\tilde{T}}-T))X_{T} + A(1, 0, 0, 0, {\tilde{T}} - T) + B(1,0,{\tilde{T}}-T) V_T+ C(1, 0,{\tilde{T}} - T)\delta _{T} + D(1, 0,{\tilde{T}} - T)\lambda _{T}$$.

#### Proposition 5

The moment generating function of $Y_T$ is given by

15 $$\begin{aligned} \textrm{E}[e^{u Y_T} | {\mathcal {F}}_t]&= e^{u A(1, 0, 0, 0, {\tilde{T}} - T)} \exp \Big ((u(1 + G(1,{\tilde{T}}-T)) + G(u(1 + G(1,{\tilde{T}}-T)), \tau ))X_t \nonumber \\&\quad + A(u(1 + G(1,{\tilde{T}}-T)), u B(1, 0, 0, 0, {\tilde{T}} - T), \nonumber \\&\quad u C(1,0,{\tilde{T}}-T), uD(1, 0,{\tilde{T}} - T),\tau ) \nonumber \\&\quad + B(u(1 + G(1,{\tilde{T}}-T)), u B(1, 0, 0, 0, {\tilde{T}} - T),\tau )V_t \nonumber \\&\quad + C(u(1 + G(1,{\tilde{T}}-T)), u C(1,0,{\tilde{T}}-T),\tau )\delta _t \nonumber \\&\quad + D(u(1 + G(1,{\tilde{T}}-T)), uD(1, 0,{\tilde{T}} - T), \tau )\lambda _t\Big ) \end{aligned}$$

#### Proof

The result is obtained from (11) substituting $u_1 = u(1 + G(1,{\tilde{T}}-T))$, $u_2 = u B(1, 0, 0, 0, {\tilde{T}} - T)$, $u_3 = u C(1,0,{\tilde{T}}-T)$ and $u_4 = uD(1, 0,{\tilde{T}} - T)$. $\square$

Given the moment generating function in (15) we get the characteristic function by substituting *u* with *iu*, then we compute $f_Y(y)$ using the COS method and recover $C_{EF}$ from (14). In the next section we tackle the problem of efficiently simulating the model in (7)–(10), while the pricing of Asian options is deferred to Section 4.

## Simulation

In this section we propose a simulation scheme for the model in (7)–(10). As will later become clear, the accuracy of the algorithm increases with the number of time discretization steps. We start by illustrating the procedure step by step, then we discuss the sources of error. Consider a set of dates $0=:t_0< t_1< \cdots < t_{n} := T$, we are interested in simulating $X_{t_j}$ for $j=0,\ldots , n$. Anyway, for sake of clarity, let us illustrate the proposed method in the case where one wants to simulate directly $\left( X_T | X_0, V_0, \delta _0, \lambda _0\right)$, omitting intermediate dates. This is without loss of generality since only simple adaptations are needed to include also the intermediate dates, as we show in Algorithm 1 where we summarize the whole simulation procedure for a generic set of dates. Given the underlying asset price trajectory is then possible to price many kind of exotic options on spot prices, including Asian options. For what concerns the price of options on futures, we note that the underlying can be simulated easily from (12) requiring only the additional simulation of $\{\delta _{t_j}\}_{j=1}^{n}$ (not required in the case of the spot price).

### Step 1: Exact simulation of $\int _{0}^{T}\lambda _s ds$ given $\lambda _0$

Using Dassios and Zhao (2013, Algorithm 3.1) we obtain a sample of the triplet $\left( N_{T}, \{\tau _k\}_{k=1}^{N_{T}}, \{\lambda _{\tau _k}\}_{k=1}^{N_{T}}\right)$, where $N_T$ is the total number of jumps in the period [0, *T*] and $\tau _k$ is the $k-$th jump time. Given the triplet we can compute

16 $$\begin{aligned} \lambda _{T}= \theta _{\lambda } + (\lambda _0 - \theta _{\lambda })e^{-k_{\lambda } T} + \sum _{k=1}^{N_T}\beta e^{-k_{\lambda }(T-\tau _k )}, \quad \int _{0}^{T} \lambda _s ds = -\frac{\lambda _T- \lambda _0 - k_{\lambda } \theta _{\lambda }T - \beta N_T}{k_{\lambda }}. \end{aligned}$$

### Step 2: Exact simulation of $\left( \delta _T, \int _{0}^{T}\delta _s ds\right)$ given $\delta _0$

Following Glasserman (2004, Section 3.3) we have

17 $$\begin{aligned} \begin{bmatrix} \delta _T\\ \int _{0}^{T}\delta _s ds \end{bmatrix} \sim {\mathcal {N}}\left( \mu _{\delta }, \Sigma _{\delta }\right) \end{aligned}$$

where

$$\begin{aligned} \mu _{\delta }&= \begin{bmatrix} (\delta _0 - \theta _{\delta })e^{-k_{\delta } T}+\theta _{\delta }\\ \theta _{\delta } T +(\delta _0-\theta _{\delta })\frac{1-e^{-k_{\delta } T}}{k_{\delta }}\end{bmatrix}, \\ \Sigma _{\delta }&= \begin{bmatrix} \frac{\sigma _{\delta }^2}{2k_{\delta }}(1-e^{-2k_{\delta } T})&{} \frac{\sigma _{\delta }^2}{2k_{\delta }^2}(1-e^{-k_{\delta } T})^2 \\ \frac{\sigma _{\delta }^2}{2k_{\delta }^2}(1-e^{-k_{\delta } T})^2 &{} -\frac{\sigma _{\delta }^2}{2 k_{\delta }^3}(1-e^{k_{\delta } T})^2 + \frac{\sigma _{\delta }^2}{k_{\delta }^2}(t - \frac{1-e^{-k_{\delta } T}}{k_{\delta }}) \end{bmatrix}. \end{aligned}$$

The transition of $\left( \delta _T,\int _{0}^{T}\delta _s ds\right)$ can thus be simulated exactly and efficiently by means of standard random numbers generators from a multivariate normal distribution.

### Step 3: Exact simulation of $\left( V_T, \int _{0}^{T}V_s ds\right)$ given $V_0$

The transition density of the terminal variance is known

18 $$\begin{aligned} V_T\overset{(\text {law})}{=}\frac{\sigma _v^{2}(1-e^{-k_v T})}{4k_v}\chi _{d}^{\prime 2}\left( \lambda \right) \end{aligned}$$

where $\chi _{d}^{\prime 2}\left( \lambda \right)$ denotes the non-central chi-squared distribution with $d:=4\theta _v k_v/\sigma _v^{2}$ degrees of freedom and non-centrality parameter $\lambda :=4k_v{e}^{{-k_v} T}V_0/\sigma _v^{2}(1-e^{-k_v T})$. Hence, $(V_T|V_0)$ is simulated exactly using standard generators from the non-central chi-squared distribution.^1^ The next step consists in simulating $\left( \int _{0}^{T} V_s ds | V_T, V_0\right)$, not a trivial problem. Broadie and Kaya (2006) develop an exact simulation scheme. Nevertheless, their proposed methodology presents several implementation issues, being generally slow to run and, despite theoretically exact, biased in practice (see e.g. Glasserman and Kim, 2011; Kienitz and Wetterau, 2012). To overcome these issues, Kyriakou et al. (Forthcoming) suggest an alternative approach based on fast computation of the moments of $\left( \int _{0}^{T} V_s ds| V_T, V_0\right)$ and subsequent random sampling from a 4-moments matched Pearson distribution. This methodology turns out to be faster and more accurate than competing benchmarks, hence, we will adopt this methodology for fast sampling $\left( \int _{0}^{T} V_s ds | V_T, V_0\right)$.^2^ Finally, we have

19 $$\begin{aligned} \int _{0}^{T}\sqrt{V_s}dW_s^v = \frac{1}{\sigma _v}\left( V_T - V_0 - k_v \theta _v T + k \int _{0}^{T}V_s ds\right) . \end{aligned}$$

### Step 4: Simulation of $X_T$ given $V_T$, $\int _{0}^{T}V_s ds$, $\delta _T$, $\int _{0}^{T}\delta _s ds$, $\lambda _T$, $\int _{0}^{T}\lambda _s ds$ and $\{\tau _k\}_{k=1}^{N_T}$

From (7) and (19):

$$\begin{aligned} X_T&= X_0 -\frac{1}{2}\int _{0}^{T} V_s ds - \mu ^{\star }\int _{0}^{T}\lambda _s ds - \int _{0}^{T}\delta _s ds - \alpha \int _{0}^{T}X_s ds + \\&\quad + \frac{\rho }{\sigma _v}\left( V_T - V_0 - k_v \theta _v T + k \int _{0}^{T}V_s ds\right) + \sqrt{1-\rho ^2}\int _{0}^{T}\sqrt{V_s}dW_x^v + \sum _{i=1}^{N_T}J_{x,i} \end{aligned}$$

where $\{J_{x,i}\}_{i=1}^{N_T}$ is a sequence of i.i.d. normal (with mean $\mu _J$ and standard deviation $\sigma _j$) random variables. Thus, we have

20 $$\begin{aligned} X_T + \alpha \int _{0}^{T}X_s \sim {\mathcal {N}}\left( {\bar{\mu }} + \sum _{i=1}^{N_T}J_{x,i}, (1-\rho ^2)\int _{0}^{T} V_s ds\right) \end{aligned}$$

where

$$\begin{aligned} {\bar{\mu }}&= X_0 -\frac{1}{2}\int _{0}^{T} V_s ds - \mu ^{\star }\int _{0}^{T}\lambda _s ds - \int _{0}^{T}\delta _s ds + \frac{\rho }{\sigma _v}\left( V_T - V_0 - k_v \theta _v T + k_v \int _{0}^{T}V_s ds\right) . \end{aligned}$$

Therefore, we can simulate $\{J_{x,i}\}_{i=1}^{N_T}$ and the quantity $X_T + \alpha \int _{0}^{T}X_s ds$ by means of standard random numbers generators from a normal distribution. Note that in case of absence of mean reversion ($\alpha =0$) the model is simulated exactly through the proposed approach. However, in presence of mean reversion, a full exact simulation scheme seems not doable. Hence, we employ a central discretization (compare with Andersen, 2008, Eq. 32):

21 $$\begin{aligned} \int _{0}^{T} X_s ds \approx T \frac{X_T + X_0}{2}. \end{aligned}$$

If we denote with *Z* the random sample from $X_T + \alpha \int _{0}^{T}X_s ds$, then we have that $X_T \approx \frac{Z - \alpha T/2 X_0}{1 + \alpha T /2}=:{\tilde{X}}_T$.

The whole simulation procedure contains two sources of error: *i*) the simulation of $\left( \int _{0}^{T}V_s ds | V_T,V_0\right)$; *ii*) the approximation in (21). The first is negligible in practice as illustrated in Kyriakou et al. (Forthcoming) and also confirmed by the numerical studies we will present in Sect. 5. The second source of error is more important. In particular, accuracy depends on the length of the interval [0, *T*] (we expect high accuracy for small values of *T*) and on the value of the parameter $\alpha$. In this case, the smaller $\alpha$ the smaller the error. We will investigate this point in the numerical section.

Finally, the asset price is computed as $S_T = S_0e^{X_T}$. In addition, it is possible to recover $\int _{0}^{T}X_s ds$ from (21) and the price of futures from (12).
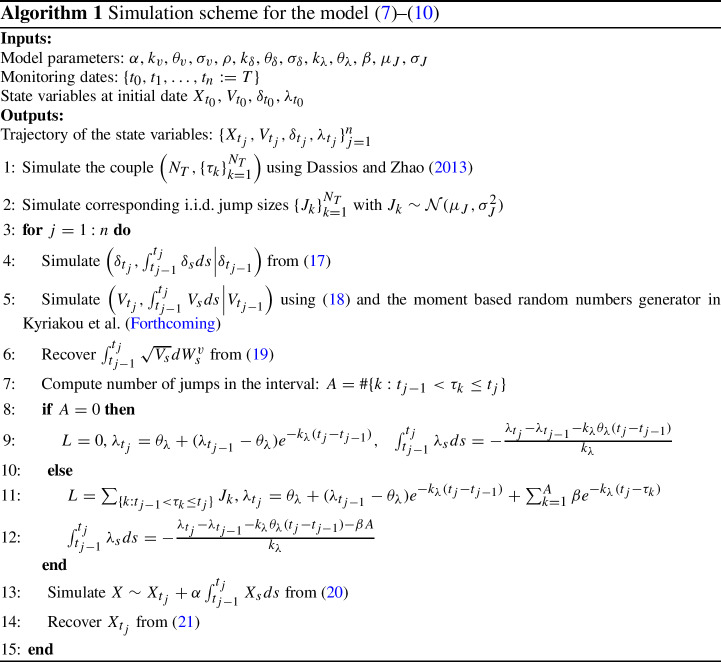


## Asian option pricing

In this section we derive formulas for the price of Asian options. The payoff depends on the average of a commodity’s spot-price. Consider the usual set of dates $0=:t_0<t_1<\cdots <t_n:=T$, then the payoff of an Asian option with strike *K* and maturity *T* is

22 $$\begin{aligned} {\mathcal {X}} = \max (0, A_{n} - K), \quad {\mathcal {Y}} = \max (0, G_{n} - K) \end{aligned}$$

where the arithmetic and geometric averages are defined respectively as $A_{n} = \frac{1}{n}\sum _{j=1}^{n} S_{t_j}$ and $G_{n} = \exp \left( \frac{1}{n}\sum _{j=1}^{n} \log S_{t_j}\right)$. This kind of option is very popular in OTC markets, especially when the underlying is the price of metal commodities (see, among others, Kaminski, 1999, Shiraya and Takahashi, 2011). In practice averaging is usually arithmetic rather than geometric. However, pricing geometric Asian options is still very important as i) this allows for exact pricing formulas and ii) the payoff of geometric averaged options is highly correlated with that of their arithmetic averaged cousins. This fact is exploited in synthetic variance reduction methods for Monte Carlo simulation. Consequently, it comes natural to use the geometric Asian option price as a control variable in a Monte Carlo simulation to obtain accurate price estimates for the arithmetic counterpart. Let’s start our discussion from the price of the geometric Asian option which is given by:

23 $$\begin{aligned} C_{G_n}&= \textrm{E}[\max (0, G_{n} - K)] = \textrm{E}\left[ \max \left( 0, S_0\exp \left( \frac{1}{n}\sum _{j=1}^{n} X_{t_j}\right) - K\right) \right] \nonumber \\&=\int _{K}^{\infty } (S_0e^{h} - K)f_{H_T}(h) dh, \end{aligned}$$

where $H_T:= \frac{1}{n}\sum _{j=1}^{n} X_{t_j}$. We derive, next, the moment generating function of $H_T$. The following proposition extends the results in Fusai and Kyriakou (2016) to the case of a mean reverting multi-factor affine model.

### Proposition 6

Define $B_0:=0$, $C_0 :=0$, $D_0:=0$, $G_0:=0$. The moment generating function of $\sum _{j=1}^{n} X_{t_j}$ is

$$\begin{aligned} \textrm{E}[e^{u \sum _{j=1}^{n} X_{t_j}}|{\mathcal {F}}_{t_0}] =e^{( n u + G_n) X_{t_0} + B_n V_{t_0} + C_n \delta _{t_0} +D_n \lambda _{t_0} + \sum _{j=1}^{n} A(ju + G_{j-1}, B_{j-1}, C_{j-1}, D_{j-1}, t_{n-j+1} - t_{n-j})}, \end{aligned}$$

where

$$\begin{aligned} B_j&:= B\left( ju+ \sum _{i=0}^{j-1}G_{i}, B_{j-1}, t_{n-j+1} - t_{n-j}\right) , \\ C_j&:= C\left( ju+ \sum _{i=0}^{j-1}G_{i}, C_{j-1}, t_{n-j+1} - t_{n-j}\right) , \\ D_j&:= D\left( ju+ \sum _{i=0}^{j-1}G_{i}, D_{j-1}, t_{n-j+1} - t_{n-j}\right) , \\ G_j&:= G\left( ju + \sum _{i=0}^{j-1}G_{i}, t_{n-j+1} - t_{n-j}\right) . \end{aligned}$$

### Proof

See Appendix E. $$\square $$

Given the moment generating function we obtain the characteristic function replacing *u* with *iu* and we price the geometric Asian option using the COS method. We turn now our attention to the pricing of the arithmetic counterpart. Kemna and Vorst (1990) first proposed to exploit the high correlation between the arithmetic and the geometric average of asset prices to reduce the variance of the Monte Carlo simulation estimator. In particular, the price of the geometric counterpart is used as control variable. We employ in our context a standard Control Variate Monte Carlo setup where, for a high accuracy, we simulate the underlying asset price process using Algorithm 1. We summarize the whole procedure in Algorithm 2 and refer to Glasserman (2004) for a detailed description of the usage of control variates method for Asian option pricing.
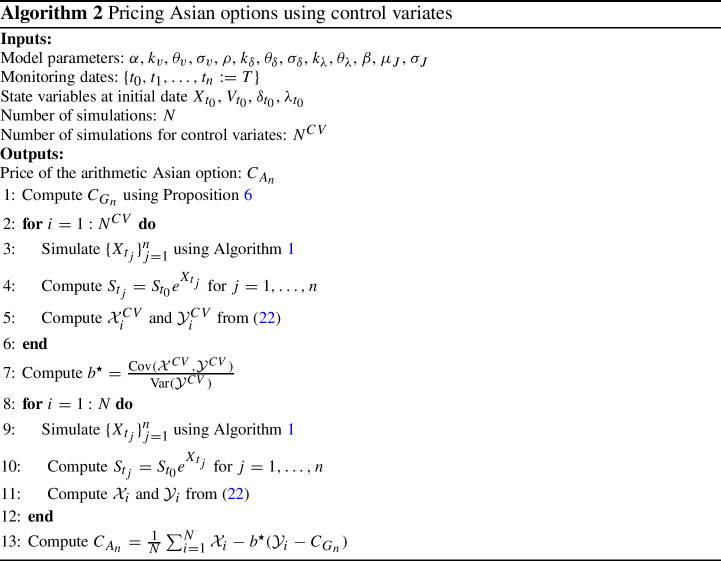


We have considered options written on the average of spot prices. Another possibility is to average futures rather than spot prices. For sake of brevity, we will not consider this case in this work. However, the payoffs of the Asian options on futures can be obtained replacing $S_{t_j}$ with $F(t_j,{\tilde{T}})$ into (22). Results obtained for the spot price can be extended to the case of Asian options on futures similarly to the case of the European options (see Proposition 5). As a final remark, we point out that is also possible to price continuously monitored geometric Asian options under the proposed model applying the method outlined in Hubalek et al. (2017, Proposition 3) and extended in Brignone and Sgarra (2020, Proposition 2).

## Numerical results

In this section we present numerical results. Before assessing the accuracy of Algorithm 1 and the pricing of Asian options we start by examining the time series of spot returns for four different commodities, namely, WTI crude oil, gold, silver and copper. We detect jumps in the historical price time series and present an econometric test which shows that jumps are not uniformly distributed over time, but tend to appear in clusters, providing further support for the proposed model. Then, we calibrate the model on real market option prices. Finally, using calibrated parameters, we evaluate the accuracy of Algorithm 1 and present numerical results on the pricing of Asian options.

All the computations are done using Matlab^®^ (Version R2021a) in Microsoft Windows 10^®^ running on a machine equipped with Intel(R) Core(TM) i7-9750HQ CPU @2.60GHz and 16 GB of RAM.

### Jump clusters in commodity prices

**Fig. 2:**
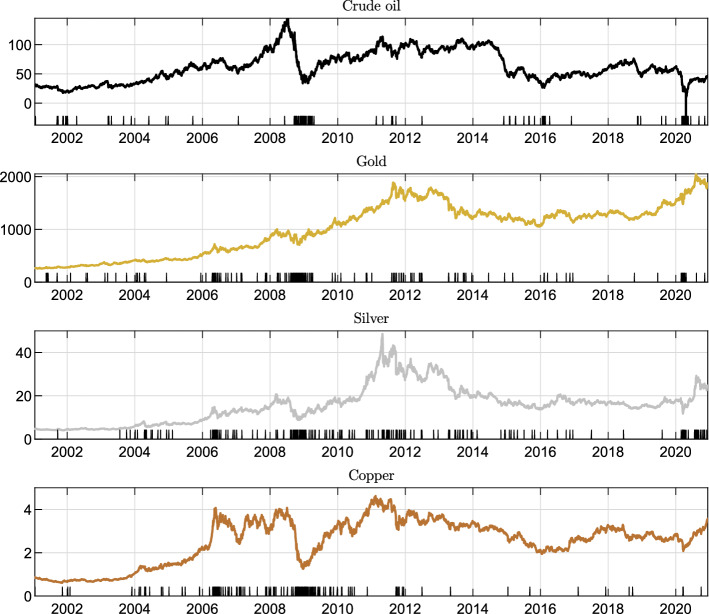
Historical time series of the spot price of four different commodities and jump occurrences (black bars)

**Fig. 3:**
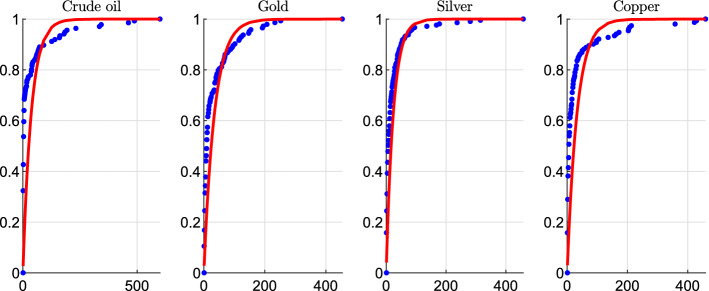
Empirical (blue points) and theoretical (red lines) cumulative distribution function (cdf) of the interarrival times for four different commodities. The theoretical cdf is the one of an exponential distribution with mean $1/\lambda$, where $\lambda$ is the daily average arrival rate (0.0285 for gold, 0.0416 for silver, 0.0271 for crude oil, 0.0303 for copper)

We illustrate and discuss the clustering effects of commodity prices, which provides us with an empirical justification for the introduction of self-excitation in the jump process. To this aim, we consider the historical time series of the spot prices from 30-Aug-2000 to 11-Dec-2020 (5048 daily observations) of four different commodities: gold, silver, crude oil, copper.^3^ The sample involves periods of crisis and financial turmoil such as the 2008 credit crisis, the summer 2011 European sovereign debt crisis, and the recent COVID-19 outbreak. Spot prices are displayed in Fig. 2 along with jump occurrences (black bars). In order to detect jumps we employ the iterated re-weighted least squares technique developed in Callegaro et al. (2017) (see also Bernis et al, 2021 for more implementation details). Jump occurrences are displayed in Fig. 2. We note that jumps are very frequent in commodity markets: we identify in the whole sample a total of 144 jumps for gold price, 210 for silver, 137 for crude oil, 135 for copper. Therefore, a model which omits the jumps in the price process is likely misspecified. We also observe that jumps appear in clusters. This is particularly evident in the case of crude oil, where we observe prolonged periods of tranquillity (e.g. we observe no jumps between 29 Jun 2012 and 26 Nov 2014) followed by periods with a lot of jumps (11 jumps in 2015). In order to show that jumps do not exhibit a constant arrival rate we propose a statistical test. If the arrival rate is constant then the jump process is a homogeneous Poisson process and the distribution of the interarrival times is exponential with mean $1/\lambda$, where $\lambda$ is the average arrival rate computed as the ratio between the total number of jumps and the number of observations in the sample (e.g. $144/5048=0.0285$ for gold). We then perform a two sample Kolmogoroff-Smirnov (KS) test, where null hypothesis is that the interarrival times are exponentially distributed with mean $1/\lambda$. The null hypothesis is rejected at the $5\%$ significance level for all the commodities considered, supporting the idea of stochastic jump intensity. In particular, for crude oil we obtain a *p*-value of 9.30E-07 which indicates a strong rejection of the hypothesis of a constant arrival rate. A graphical illustration is provided in Fig. 3 where we compare the empirical distribution of the interarrival times with the theoretical exponential distribution. If the arrival rate was constant then the two cdfs would match, but we note that this is not the case. The choice of using an Hawkes process to model the jump intensity (which is standard in the literature on equity price modeling) allows to take into account this feature of commodity returns.

### Calibration

**Fig. 4:**
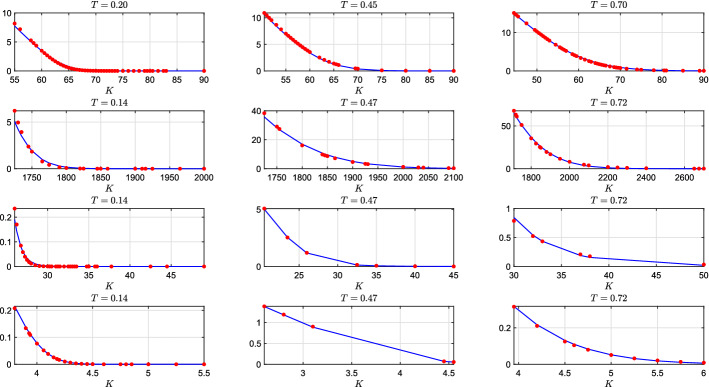
Market (red points) and model (blue lines) implied European call option on futures prices on 3 Mar 2021 for four different commodities: crude oil (top subplots), gold (second line), silver (third line) and copper (bottom subplots)

We calibrate the proposed model (7)–(10) on observed market option prices. In commodity markets the most liquid quoted options are American options. Hence, for a given date (Friday 5 March 2021) we collect American option prices for four different commodities: crude oil WTI, gold, silver and high grade copper.^4^ We consider three different maturities for each commodity ranging from 1 to 9 months (options with longer maturities are not liquid enough). In order to avoid liquidity problems, we keep all the contracts whose trading volume at the end of the day is greater than two. We end up with 125 options for crude oil WTI, 57 for gold, 44 for silver and 38 for copper. Then, we convert American option prices to Europeans. Following Trolle and Schwartz (2009) we compute the implied volatility from American option prices using the Barone-Adesi and Whaley (1987) formula, then, we calculate European call options on futures prices using the Black (1976) formula. Hence, we can calibrate the model by matching market and model implied prices of European call options (on futures). Model implied prices are computed from (14), for implementing the COS method infinite summations are truncated at the $2^9$-th element and ODEs are solved numerically using an explicit Runge-Kutta (4,5) formula.^5^ More precisely, we solve the following optimization problem:

$$\begin{aligned} \min _{\Theta }\frac{1}{{\tilde{n}}_{K}}\frac{1}{{\tilde{n}}_{T}} \sum _{t=1}^{{\tilde{n}}_{T}}\sum _{k=1}^{{\tilde{n}}_{K}} \frac{(C^{\text {Mkt}}_{t,k} - C^{\Theta }_{t,k})^2}{C^{\text {Mkt}}_{t,k}} \end{aligned}$$

where $\Theta$ is the vector of parameters, ${\tilde{n}}_{K}$ is the number of strikes, ${\tilde{n}}_{T}$ is the number of maturities, $C^{\text {Mkt}}_{t,k}$ is the market price of the European call option on future while $C^{\Theta }_{t,k}$ is the model price. We impose standard constraints: $\{V_0, \lambda _0, \alpha , \sigma _J, k_v, \theta _v, \sigma _v, k_{\delta }, \sigma _{\delta }, k_{\lambda }, \theta _{\lambda }, \beta \}>0$, $-1<\rho <1$ and $k_{\lambda }>\beta$. In order to mitigate the dependence on the initial point supplied to the optimizer we randomly generate 5000 model parameters, then we evaluate the objective function in each of them and take the 10 parameters combinations with smallest objective function (the total running time for this step in our PC is around 11 minutes). Next, we run 10 different optimizations starting from those points (optimization is performed using the built in Matlab^®^ function fmincon with the interior point algorithm and the time required for this step is around 1 hour, i.e. nearly 6 minutes for each starting point). Finally, we take those parameters where the objective function presents the smallest value. The results of this procedure are reported in Table 1, where we show calibrated parameters, and in Fig. 4, where we compare the model and market option prices. Now, some comments are in order. The parameter controlling the mean reversion $\alpha$ is smaller for crude oil than the other commodities. This is consistent with literature on oil modeling. Indeed, despite mean reversion in commodity markets is a widely acknowledged stylized feature, for the specific case of oil, many authors started excluding mean reversion from price dynamics (Trolle and Schwartz, 2009; Larsson and Nossman, 2011; Shiraya and Takahashi, 2011; Cortazar et al., 2017). In particular Larsson and Nossman (2011) find no evidence of autocorrelation of log-returns from 25-May-1989 to 25-May-2009. Meade (2010) finds better out of sample performances for models without mean reversion. On the other hand, we find strong mean reversion in the copper price, consistently with Schöne and Spinler (2017). For what concerns the parameters governing the volatility process, we find that the volatility of crude oil returns is much less persistent than other commodities as witnessed by the higher value of $k_v$. Moreover, we find that the Feller condition ($2k_v \theta _v>\sigma _v^2$) is respected for silver and copper but not for crude oil and gold. Note that this does not indicate that the model is misspecified for crude oil and gold. Indeed, when option pricing models with CIR-type variance (e.g. Heston model) are calibrated on real option quotes, it often happens that the Feller condition is not satisfied (see e.g. Rouah, 2013, Table 6.2). The violation of the Feller condition implies the introduction of a non-negligible bias when pricing options via simulation with the Euler scheme or other similar methods (see the discussion in Begin et al., 2015). This problem is not relevant to our simulation approach outlined in Sect. 3 (see Sect. 5.3). Indeed, in our approach we simulate the variance process directly according to a non central chi-squared distribution which is always positive also when the Feller condition is not respected (see also Broadie and Kaya, 2006). The parameter $\rho$, which controls the leverage effect, is negative across all the commodities. This means that when the prices drop, volatility rises. The leverage effect is consistent with the phenomenon in which many investors hedge their physical risks with forward contracts. As a result, panic can break out when prices drop, pushing volatility up. Anyway, we find that this effect is much more pronounced for crude and gold than silver and copper. Regarding the convenience yield dynamics, all commodities display a similar degree of persistence. The long run mean is positive for oil and copper and negative for gold and silver. The diffusion coefficients have comparable magnitude, except for that of silver which is smaller. Finally, regarding the parameters of the jump intensity, we find that the parameter controlling the self-exciting effect $\beta$ is similar among different commodities and the expected number of jumps per year (computed according to Dassios and Zhao, 2013, Proposition 2.3) is 5.98 for Crude oil, 4.35 for Gold, 6.10 for Silver and 7.26 for Copper.

**Table 1:** Calibrated parameters for the model in (7)–(10) on commodity option quotes

$\Theta$	Crude oil	Gold	Silver	Copper
$V_0$	0.0242	0.0057	0.0035	0.0051
$\delta _0$	0.1103	0.0833	0.0915	$-$0.0164
$\lambda _0$	7.2448	4.6698	5.1943	6.3883
$\alpha$	0.0637	0.0822	0.1511	0.2166
$\mu _J$	$-$0.0099	$-$0.0130	$-$0.0056	$-$0.0052
$\sigma _J$	0.0296	0.0163	0.0152	0.0156
$\rho$	$-$0.7163	$-$0.9136	$-$0.0501	$-$0.0619
$k_v$	6.7272	0.8697	0.9139	2.7416
$\theta _v$	0.0175	0.1746	0.2493	0.0520
$\sigma _v$	0.6872	0.9176	0.6315	0.2574
$k_{\delta }$	0.7418	0.0667	1.8986	0.8709
$\theta _{\delta }$	0.1674	$-$0.1225	$-$0.1526	0.1065
$\sigma _{\delta }$	0.4424	0.2420	0.0542	0.4271
$k_{\lambda }$	8.8334	12.2181	8.8138	9.7457
$\theta _{\lambda }$	3.8283	2.0689	4.2062	4.5081
$\beta$	2.9290	3.2874	2.9650	2.7138

Finally, since there is no closed solution for *A*, *B* and *D* in Proposition (4), we display the values of $F_1$, $F_2$ and $F_3$ together with the functions *A*,*B*,*C*,*D*, and *G* for a different values of $u_1$ ranging between 0 and 20, $u_2=u_3=u_4=0$ and different times $\tau$.

**Fig. 5:**
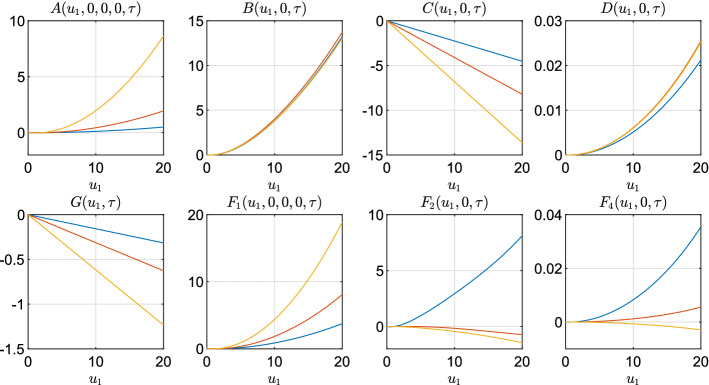
Blue, red and yellow lines correspond to, respectively, $\tau = 1/4$, $\tau = 1/2$ and $\tau = 1$

### Accuracy of Algorithm 1 and Asian option pricing

In order to assess accuracy of the proposed model simulation scheme we follow the procedure outlined in Cai et al. (2017, Section 4). First, we need to estimate the error we are committing when simulating the model (7)–(10) using Algorithm 1. To this aim, we consider the problem of pricing an at the money (ATM) European call option on spot (for simplicity and facilitation of error comparisons, let us assume an initial price for the underlying equal for all the commodities $S_0=100$), then we compare the true option price computed from (13) using the COS method and the price of the same option obtained through a high number of simulations (following Broadie and Kaya, 2006 we use $10^9$ simulations to get an accuracy at least to the fourth decimal place). The bias is the difference between the two values. Results are reported in Table 2. From this table, we can appreciate how the accuracy drastically increases with the number of time steps *n*. Moreover, for $n=16$ we find that the bias is close to 0, meaning that if one increases further *n* the error will disappear. This finding also confirms that the error coming from the simulation of $\left( \int _{0}^{T} V_s ds| V_T,V_0\right)$ is negligible in practice. The speed of mean reversion $\alpha$ impacts on the accuracy: for crude oil we have $\alpha =0.0637$ and in this case we register the best performances of Algorithm 1, with a bias smaller (in absolute value) than 0.01 for $n=2$. When $\alpha$ increases we need higher *n* to reach similar accuracy, for example, in the case of silver, we need $n=8$ for an absolute bias smaller than 0.01.

**Table 2:** Performances of Algorithm 1. Model parameters as in Table 1. True prices of the European call options with $S_0 = K = 100$ and $T=1$: 4.8675 (crude oil), 5.7837 (gold), 13.1202 (silver), 9.1293 (copper)

*n*	1	2	3	4	6	8	12	16
Crude oil								
Price	4.8341	4.8595	4.8640	4.8657	4.8670	4.8672	4.8673	4.8675
Bias	$-$0.0335	$-$0.0081	$-$0.0036	$-$0.0019	$-$0.0006	$-$0.0004	$-$0.0003	$-$0.0001
s.e.	3.43E$-$04	3.45E$-$04	3.45E$-$04	3.45E$-$04	3.45E$-$04	3.45E$-$04	3.45E$-$04	3.45E$-$04
Gold								
Price	5.6942	5.7604	5.7737	5.7777	5.7808	5.7820	5.7832	5.7833
Bias	$-$0.0895	$-$0.0233	$-$0.0100	$-$0.0061	$-$0.0029	$-$0.0017	$-$0.0005	$-$0.0004
s.e.	3.14E$-$04	3.17E$-$04	3.18E$-$04	3.18E$-$04	3.18E$-$04	3.18E$-$04	3.18E$-$04	3.18E$-$04
Silver								
Price	12.7451	13.0238	13.0767	13.0961	13.1089	13.1139	13.1170	13.1195
Bias	$-$0.3751	$-$0.0965	$-$0.0435	$-$0.0241	$-$0.0113	$-$0.0063	$-$0.0032	$-$0.0007
s.e.	6.82E$-$04	6.98E$-$04	7.01E$-$04	7.02E$-$04	7.02E$-$04	7.03E$-$04	7.03E$-$04	7.03E$-$04
Copper								
Price	8.9835	9.0934	9.1130	9.1198	9.1257	9.1265	9.1282	9.1292
Bias	$-$0.1458	$-$0.0359	$-$0.0163	$-$0.0096	$-$0.0036	$-$0.0028	$-$0.0011	$-$0.0001
s.e.	4.95E$-$04	5.03E$-$04	5.04E$-$04	5.04E$-$04	5.05E$-$04	5.05E$-$04	5.05E$-$04	5.05E$-$04

**Fig. 6:**
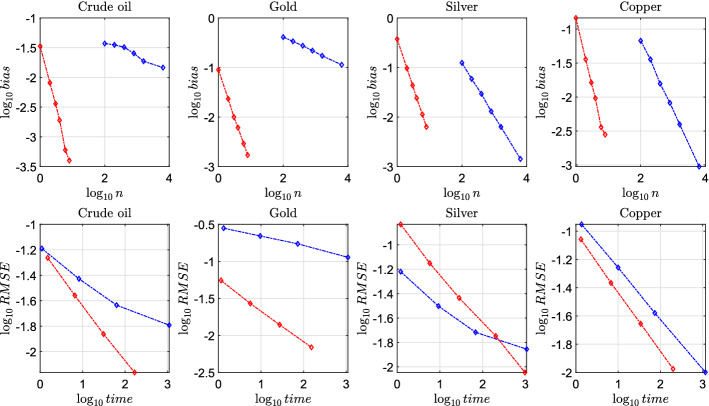
Performances of Algorithm 1 (red line) and Euler scheme (blue line) for the problem of pricing an European call option under limited computational budget for the model in (7)–(10) by simulation. Top subplots present the relationship between bias and *n*, while bottom subplots present the relationship between RMSE and computing time in seconds (in log-log scale). Parameters are as in Table 1. Other parameters: $S_0 = 100$, $K = 100$ and $T=1$. Further notes: see Table 2

Having confirmed that accuracy increases with the number of time steps *n*, the next step is to study the performances of Algorithm 1 in terms of trade-off between accuracy (measured through the Root mean Squared Error, $\text {RMSE} = \sqrt{\text {bias}^2 + \text {standard error}^2}$, see Li and Wu, 2019 for more details) and computational efficiency (running time expressed in seconds). Following standard literature (e.g. Broadie and Kaya, 2006, Cai et al., 2017, Li and Wu, 2019) we report the performances of the Euler scheme for benchmark comparison. First of all, it is worth noting that the proposed simulation method differs greatly from discretization methods like the Euler scheme. Indeed, we just need to split the time horizon into a very small number of time discretization steps (indeed, biases are very close to 0 for $n=16$), while Euler scheme uses simple but rough approximations which work well only on small time steps, with the natural consequence that Euler scheme needs a higher number of time steps to achieve a level of accuracy similar to that of our proposed approach. However, when using Algorithm 1 and the Euler scheme for the purpose of pricing options with a limited computational budget, a trade–off is intrinsically established between increasing the number of time steps *n* (to reduce the bias) and the number of simulations *N* (to reduce the statistical error). Increasing *n* will improve accuracy but also increase the computational cost. We compute the bias of the Euler scheme using $10^9$ simulations for different number of time discretization steps $n=\{200, 400, 800, 1600, 6400\}$. The behavior of the bias of both Algorithm 1 and the Euler scheme for different time steps is reported in log-log scale in the top panel of Fig. 6. From this figure we can appreciate how the bias of Algorithm 1 decreases faster than that of the Euler scheme. More precisely, by regressing $\log _{10} (bias)$ vs $\log _{10} (n)$ we get a slope around $-2$ (for all parameter sets) for Algorithm 1 and around $-0.9$ for the Euler scheme, implying that the bias of our proposed simulation method is approximately proportional to $1/(n^2)$, while the bias of the Euler scheme is approximately proportional to 1/*n*. Duffie and Glynn (1995) show that if the bias decays at the order of $1/(n^p)$, then one should increase *n* proportionally to $N^{1/(2p)}$ to achieve asymptotic optimality. Hence, it is possible to improve efficiency of the proposed method with a smaller computational effort on the bias reduction with respect to the Euler scheme: having estimated $p \approx 2$ for our Algorithm 1 we can select *n* increasing at the order $N^{1/4}$, while in the case of the Euler scheme, since $p \approx 1$, *n* should be proportional to $N^{1/2}$. The trade-off between accuracy and computational efficiency in a log-log scale is shown in the bottom panel of Fig. 6 while numerical values of RMSE and running times are reported (along with biases) in Table 3. Results show that the RMSE of the proposed simulation method decays faster than the Euler scheme. In particular, we get the following convergence rate of the RMSE for Algorithm 1: 0.44 for crude oil, 0.43 for gold, 0.44 for silver and 0.42 for copper. These are only slightly smaller than those of a theoretically unbiased estimator (which would be around 0.5, see e.g. Broadie and Kaya, 2006) but higher than those of the Euler scheme, which are around 0.30 for all the parameter sets considered, confirming the superior performances of the proposed approach with respect to the benchmark. In Fig. 6, in the case of silver we also included the case with $n=8$ and $N=1024\times 10^4$ for Algorithm 1 to better highlight its superior performances. Another important aspect in the comparison between the proposed approach and the Euler scheme is that in the latter the discretization of the variance process may generate negative values in intermediate steps with a significant probability when the Feller condition $2k_v\theta _v>\sigma _v^2$ is violated (this is the case of the calibrated parameters for crude oil and gold). This brings extra error in the simulation procedure, explaining the poor performances of the Euler scheme in the case of gold (where, in addition, the initial value of the variance process $V_0$ is very small, increasing the probability of the variance process reaching zero).

**Table 3:** Speed–accuracy comparison between Algorithm 1 and Euler scheme. Further notes: see Fig. 6

*N*	Euler				Algorithm 1			
	*n*	Bias	RMSE	Time	*n*	Bias	RMSE	time
Crude oil								
$10^4$	200	0.0353	0.1144	0.15	1	0.0335	0.1167	0.46
$4\times 10^4$	400	0.0322	0.0646	1.10	2	0.0081	0.0546	1.50
$16\times 10^4$	800	0.0254	0.0373	8.21	3	0.0036	0.0276	6.54
$64\times 10^4$	1600	0.0187	0.0232	62.90	4	0.0019	0.0137	31.04
$256\times 10^4$	6400	0.0146	0.0161	1093.85	6	0.0006	0.0069	166.98
Gold								
$10^4$	200	0.3375	0.3547	0.19	1	0.0895	0.1339	0.24
$4\times 10^4$	400	0.2752	0.2806	1.32	2	0.0233	0.0553	1.15
$16\times 10^4$	800	0.2187	0.2204	9.55	3	0.0100	0.0270	5.59
$64\times 10^4$	1600	0.1721	0.1726	72.84	4	0.0061	0.0140	27.35
$256\times 10^4$	6400	0.1132	0.1134	1100.87	6	0.0029	0.0069	152.68
Silver								
$10^4$	200	0.0586	0.1183	0.19	1	0.3751	0.4312	0.26
$4\times 10^4$	400	0.0294	0.0603	1.21	2	0.0965	0.1472	1.21
$16\times 10^4$	800	0.0130	0.0315	9.24	3	0.0435	0.0707	5.79
$64\times 10^4$	1600	0.0063	0.0192	68.37	4	0.0241	0.0367	28.17
$256\times 10^4$	6400	0.0014	0.0139	1071.30	6	0.0113	0.0179	202.02
Copper								
$10^4$	200	0.0358	0.2247	0.20	1	0.1458	0.2141	0.34
$4\times 10^4$	400	0.0157	0.1118	1.36	2	0.0359	0.0876	1.32
$16\times 10^4$	800	0.0082	0.0553	9.98	3	0.0163	0.0431	6.75
$64\times 10^4$	1600	0.0040	0.0264	73.44	4	0.0096	0.0221	34.03
$256\times 10^4$	6400	0.0009	0.0100	1160.33	6	0.0036	0.0106	199.52

Finally, given model parameters and having tested the accuracy of the simulation scheme, we consider the problem of pricing an Asian option using Algorithm 2. We assume an initial price $S_0=100$ and compute the price of European and Asian call options (geometric and arithmetic averaging) using (13) and (23) with discrete monitoring (12 monitoring dates) and several strikes. ODEs are solved numerically using an explicit Runge-Kutta (4,5) formula, while infinite summations for the implementation of the Fourier-Cosine method are truncated at the $2^{10}$ element. Algorithm 2 is implemented with $N=10^6$ and $N^{CV}=10^4$ simulations. Results are reported in Table 4. European call option price is computed in approximately 1 second, the geometric Asian option price is computed in around 8 seconds, while the price of the arithmetic counterpart is obtained in around 110 seconds for all the parameter sets. The usage of the geometric Asian option price as control variable turns out to be extremely useful, allowing for a variance reduction around 99% across all the parameter sets, maturities and strikes.

**Table 4:** European (computed as in formula 13) and discretely monitored (12 monitoring dates) geometric (23) and arithmetic Asian call option prices (Algorithm 2) for the four commodities. Model parameters as in Table 1, other parameters: $S_0 = 100$, $T=1$, $N_{CV} = 10^4$, $N=10^6$, $n=12$

*K*	European	Geometric	Arithmetic (s.e.)	European	Geometric	Arithmetic (s.e.)
	Crude oil			Gold		
90	8.6404	6.9602	7.1203 (2.24E−04)	10.8826	8.2583	8.4455 (2.53E−04)
100	4.8676	2.3543	2.4421 (1.53E−04)	5.7837	2.3714	2.4660 (1.42E−04)
110	2.5663	0.5438	0.5951 (1.24E−04)	2.6372	0.2719	0.3116 (9.47E−05)
	Silver			Copper		
90	19.1242	11.5627	12.1210 (8.42E−04)	14.3348	10.6565	10.9694 (3.58E−04)
100	13.1202	5.1451	5.5849 (7.40E−04)	9.1293	4.4929	4.7173 (2.84E−04)
110	8.7192	1.8528	2.1673 (6.59E−04)	5.5327	1.4246	1.5782 (2.47E−04)

## Conclusions

In this paper we propose a new model for pricing commodity options. The model accounts for mean reversion, stochastic convenience yield, stochastic volatility and stochastic jump intensity. For the latter, we provide empirical evidence of self-excitation across four different commodity markets. After presenting the model under the historical measure, we introduce a structure preserving change of measure and describe the model under a risk–neutral measure. Then, by calibrating the proposed model on real market option prices, we find an excellent fit. We develop an efficient simulation scheme for the proposed model, we identify sources of error and present a comparison with the classic Euler scheme. Finally, we derive semi-closed formulas for the price of geometric Asian options under the proposed model and combine this result with the simulation scheme to develop a Control Variate simulation strategy for accurate evaluation of arithmetic Asian option prices, which are very popular derivative instruments in commodity markets. The methodology is able to provide accurate results at a reasonable computational cost and the simulation scheme can be used for pricing other path-dependent derivatives written on commodity prices.

## Appendix A. Proof of Proposition 1 (Some hints)

In this section we outline the proof of existence and uniqueness of the solution of the system of stochastic differential equations describing the model investigated in the present paper. We write the equations again for ease of reading:

A.1 $$\begin{aligned} dX_t&= \left( \mu - \frac{V_t}{2} - \lambda _t \mu ^{*}- \delta _t - \alpha X_t \right) dt + \sqrt{V_t}dW^{x}_t + J_x dN_t, \end{aligned}$$

A.2 $$\begin{aligned} dV_t&= k_v (\theta _v-V_t)dt + \sigma _v\sqrt{V_t}dW^{v}_t,\end{aligned}$$

A.3 $$\begin{aligned} d \delta _t&= k_{\delta }(\theta _{\delta } - \delta _t)dt + \sigma _{\delta } dW^{\delta }_t \end{aligned}$$

A.4 $$\begin{aligned} d\lambda _t&= k_{\lambda }(\theta _{\lambda }-\lambda _t)dt + \beta dN_t, \end{aligned}$$

where all the notations have been defined in Sect. 2. We start by focusing on the last equation, which raises the most critical issues, involving the dynamics of the intensity of jumps of a Hawkes process. Existence and uniqueness for this equation has been proved in different ways and several generalization of the basic results are available in the literature, see Brémaud and Massoulié (1996) and Morariu-Patrichi and Pakkanen (2018). The easiest way to prove existence and uniqueness of Hawkes processes is based on the cluster representation provided by Hawkes and Oakes (1974). This is a constructive method which allows to obtain a Hawkes process as a linear superposition of Galton-Watson branching processes and, by this approach, these authors were the first to discuss existence and uniqueness of a strong solution of (A.4) for the stationary case. The proof of existence and uniqueness, under a non-explosion condition, for the nonstationary (and nonlinear) case can be found in Massoulié (1998) (Theorem 1, pag.6), where the Poisson Embedding method is exploited.

An alternative proof of existence and uniqueness of solution for (A.4) can be provided by using the well-known Dawson and Li (2012) representation of Continuous Branching Processes with Immigration; this approach can be applied to the present case since only positive jumps (all of the same size $\beta$) appear in the driving term. This approach has been exploited in Bernis et al. (2021) for an equation of similar type.

As a second step we can write (A.1) and (A.2) by decomposing the correlated Wiener processes $dW^{x}$ and $dW^{v}$ into the linear combination of two orthogonal Wiener processes in the following way:

A.5 $$\begin{aligned} dX_t&= \left( \mu - \frac{V_t}{2} - \delta _t - \alpha X_t \right) dt + \sqrt{V_t}[\rho dW^{1}_t + \sqrt{1 - \rho ^2} dW^{2}_t] + \bar{J_x} d \bar{N_t}, \end{aligned}$$

A.6 $$\begin{aligned} dV_t&= k_v (\theta _v-V_t)dt + \sigma _v\sqrt{V_t}dW^{2}_t, \end{aligned}$$

where $\bar{J_x} d \bar{N_t}$ denotes the non-compensated jump process. By this way, (A.6) becomes independent of the others. The last equation, describing the volatility dynamics, is the classical equation of the CIR type, for which existence and uniqueness of a strong solution can be proved by applying Yamada-Watanabe theorem (see Karatzas and Shreve, 1991, Proposition 2.13, Chapter 5, p. 291). As a continuous process, on every interval [0, *T*] it will be bounded.

(A.3) is an Ornstein-Uhlenbeck stochastic differential equation driven by a Wiener process and existence and uniqueness of the solution of this equation is a classical result of stochastic calculus. As a continuous process, on every interval [0, *T*] it will be bounded as well.

Finally, we can focus on (A.5), describing the log–returns dynamics. By performing an equivalent change of measure with the following likelihood process:

$$\begin{aligned} L_t := {\mathcal {E}} \left\{ \int _0^t (\lambda _{s-} -1 ) (dN_s - ds)\right\} , \end{aligned}$$

the jump term appearing in (A.5) turns into a Compound Poisson process with constant intensity. We point out that this measure change involves only the jump term, since all the other terms are assumed to be independent on the jump part. Moreover the measure change will establish a one-to-one mapping between the processes under the two measures. The likelihood process $L_t$, under the assumption $\textrm{E}^{{\mathbb {P}}} [e^{J_x}]$ can be proved to be a true martingale since $\textrm{E}^{{\mathbb {P}}} [\int _0^t \lambda _s ds ] < + \infty , \forall t \in [0,T]$. (A.5) is now a linear stochastic differential equation driven by a Lévy process, and the classical result of existence and uniqueness of strong solutions holds (a proof can be found in Applebaum (2004, Theorem 6.2.3, p. 304), since the boundedness of both $\delta _t$ and $V_t$ on the time interval [0, *T*] ensures the required Lipschitz conditions.

## Appendix B. Proof of Proposition 2

We extend to the present modelling framework the results in Zhang et al. (2009) (in our case, we have a vector Hawkes process and the process $J_x dN_t$ driving the log-returns dynamics is marked). Define $m_t := \ln {M_t}$; then

B.1 $$\begin{aligned} dm_t&= \kappa _1 (\xi ) k_{\lambda } (\theta _{\lambda } - \lambda _t ) dt + \kappa _2 (\xi ) dt + \kappa _1 (\xi ) \beta dN_t + \xi J_x dN_t - \frac{1}{2} \phi ^2_x (t) dt -\phi _x (t) dW^x_t\nonumber \\&\quad - \rho V_t \phi _x (t) \phi _v (t) dt - \frac{1}{2} \phi ^2_v (t) dt - \phi _v (t) dW^v_t - \frac{1}{2} \phi ^2_{\delta } (t) dt - \phi _{\delta } (t) dW^{\delta }_t. \end{aligned}$$

By applying Itô lemma to $M_t$ we obtain:

$$\begin{aligned} dM_t&= M_t dm_t + \frac{1}{2} M_t d [m_t , m_t]^c + M_t \int _{-\infty }^{+\infty } [\exp {(\kappa _1 (\xi ) \beta + \xi z)} \\&\quad - 1 - (\kappa _1 (\xi ) \beta + \xi z )] F(dz) dN_t, \end{aligned}$$

where $[m_t , m_t]^c$ denotes the quadratic variation of the continuous component of $m_t$ and $F(\cdot )$ denotes the random measure of the jumps size *J* and $\lambda _t \nu (dz) dt$ denotes the predictable compensator of the process $J_x dN_t$. We can write the last equation in the following form:

$$\begin{aligned} dM_t&= M_t [\kappa _1 (\xi ) k_{\lambda } ( \theta _{\lambda } - \lambda _t ) dt + \kappa _2 (\xi ) dt + (\kappa _1 (\xi ) \beta + \xi z F(z) ) dN_t ] \\&\quad + M_t \Big [ - \frac{1}{2} \phi ^2_x (t) dt - \phi _x (t) dW^x_t -\frac{1}{2} \phi ^2_v (t) dt - \phi _v (t) dW^v_t \\&\quad - \frac{1}{2} \phi ^2_{\delta } (t) dt - \phi _{\delta } (t) dW^{\delta }_t - \phi _x (t) \phi _v (t) \rho V_t dt \Big ] + \frac{1}{2} M_t d [m_t , m_t]^c \\&\quad + M_t \int _{-\infty }^{+\infty } [\exp {(\kappa _1 (\xi ) \beta + \xi z)} - 1 - (\kappa _1 (\xi ) \beta + \xi z )] F(dz) dN_t, \end{aligned}$$

which, after the introduction of the compensator of the jump process, can be written as

$$\begin{aligned} dM_t&= M_t \kappa _1 (\xi ) k_{\lambda } \theta _{\lambda } dt + M_t \kappa _2 (\xi ) dt - M_t \lambda _t \kappa _1 (\xi ) k_{\lambda }- M_t \phi _x (t) dW^x_t - M_t \phi _v (t) dW^v_t +\\&\quad - M_t \phi _{\delta } (t) dW^{\delta }_t - M_t \lambda _t \int _{-\infty }^{+\infty }[\exp {(\kappa _1 (\xi ) \beta + \xi z)} - 1 ] \nu (dz) dt+\\&\quad + M_t \int _{-\infty }^{+\infty } [\exp {(\kappa _1 (\xi ) \beta + \xi z)} - 1] [F(dz) dN_t - \lambda _t \nu (dz) dt], \end{aligned}$$

since $$\frac{1}{2} M_t d [m_t , m_t]^c = [\frac{1}{2} \phi ^2_{x} (t) + \frac{1}{2} \phi ^2_{v} (t) + \frac{1}{2} \phi ^2_{\delta } (t) + \phi _x (t) \phi _v (t)\rho V_t ] M_t dt $$. The integral with respect to the compensated jump measure $$F(dz) dN_t - \lambda _t \nu (dz) dt$$ is a local martingale as the integrals with respect to the Wiener processes $$dW^x_t, dW^v_t , dW^{\delta }_t$$, so $$M_t $$ is a local martingale if and only if (6) hold. In order to prove that the likelihood process $$\frac{d{\mathbb {Q}}}{d{\mathbb {P}}} \big |_{{\mathcal {F}}_t} = \frac{M_t (\xi , \phi _x , \phi _{\delta } , \phi _v )}{ M_0 (\xi , \phi _x , \phi _{\delta } , \phi _v )} $$ is a true martingale with $$\textrm{E}\left[ \frac{d{\mathbb {Q}}}{d{\mathbb {P}}} \big |_{{\mathcal {F}}_t} \right] = 1$$, we need to apply the uniform integrability criterion proposed by Sokol and Hansen (2015), which in this case holds thanks to the non-explosion condition (5).

By the previous construction, it follows that the moment-generating function under ${\mathbb {Q}}$ is given by the ratio: $\psi ^{{\mathbb {Q}}} (z) = \frac{\exp { (\kappa _1 (\xi ) \beta )} \psi (z + \xi )}{\exp { (\kappa _1 (\xi ) \beta )} \psi (\xi )}$, from which the result follows.

## Appendix C. Proof of Proposition 3

The conditional moment generating function of $\lambda$ under ${\mathbb {Q}}$ is

$$\begin{aligned} \textrm{E}^{{\mathbb {Q}}} [ \exp {( u \lambda ^{{\mathbb {Q}}}_T )} |{\mathcal {F}}_t ]&= \exp {(- m_t )} \textrm{E}^{{\mathbb {P}}} \left[ \exp {( m_T + u e^{ \kappa _1 (\xi ) \psi (\xi ) \beta } \lambda ^{{\mathbb {P}}}_T )} |{\mathcal {F}}_t \right] . \end{aligned}$$

By denoting by $f(t,u, \lambda , m) = \textrm{E}^{{\mathbb {P}}} \left[ \exp {( m_T + u e^{ \kappa _1 (\xi ) \beta } \lambda _T^{{\mathbb {P}}} )} |{\mathcal {F}}_t \right]$ the conditional expectation, which is a martingale by definition, by applying Itô lemma and by imposing a vanishing condition on the drift, we obtain the following PIDE:

C.1 $$\begin{aligned}&\frac{\partial f}{\partial t} + [\kappa _1 (\xi ) k_{\lambda } (\theta _{\lambda } - \lambda ) + \kappa _1 (\xi )] \frac{\partial f}{\partial m}+ k_{\lambda } (\theta _{\lambda } - \lambda ) \frac{\partial f}{\partial \lambda } \end{aligned}$$

C.2 $$\begin{aligned}&\quad +\lambda \int _{-\infty }^{+\infty } [ f(t, \lambda + \beta , m + \kappa _1 \beta + \xi z )-f(t,\lambda , m)]\nu (dz)]\nonumber \\&\quad - \frac{1}{2} [ \phi ^2 _x + \phi ^2 _v + \phi ^2 _{\delta } + 2 \rho V \phi _x \phi _v ] \frac{\partial f}{\partial m} + \frac{1}{2} [ \phi ^2 _x + \phi ^2 _v + \phi ^2 _{\delta } + 2 \rho V \phi _x \phi _v ] \frac{\partial ^2 f}{\partial m^2} =0. \end{aligned}$$

If we guess a solution of the following form:

$$\begin{aligned} f(t, \lambda _t , m_t ) = \exp { [ A(t,T) + e^{ \kappa _1 (\xi ) \psi (\xi ) \beta } B(t,T) \lambda _t + C(t,T) m_t] }, \end{aligned}$$

with terminal conditions $$ A(T,T)=0, B(T,T)= w, C(T,T)=1$$, and denote by $$\epsilon =e^{ \kappa _1 (\xi ) \beta } \psi (\xi ) $$, we can write:

$$\begin{aligned} \frac{\partial f}{\partial t} = \left[ \frac{\partial A}{\partial t} + \epsilon \lambda _t \frac{\partial B}{\partial t} + m_t \frac{\partial C}{\partial t} \right] f, \frac{\partial f}{\partial m} = Cf, \frac{\partial ^2 f}{\partial m^2} = C^2 f, \frac{\partial f}{\partial \lambda } = \epsilon Bf. \end{aligned}$$

By inserting these expressions into (C.2) we obtain the following equation, that must be satisfied for every value of $\lambda , m$:

$$\begin{aligned}&\frac{\partial A}{\partial t} + [ \epsilon k_{\lambda } \theta _{\lambda } B + k_{\lambda } \theta _{\lambda } \kappa _1 (\xi ) C + \kappa _2 (\xi ) C ] + m_t \frac{\partial C}{\partial t} \\&\quad + \lambda _t \left[ \epsilon \frac{\partial B}{\partial t} - \kappa _1 (\xi ) k_{\lambda } C - \epsilon k_{\lambda } B + \int _{-\infty }^{+\infty } [ e^{ C \kappa _1 (\xi ) \beta + B \epsilon \beta + \xi z } -1 ] d\nu (z) \right] \\&\quad - \frac{1}{2} [ \phi ^2 _x + \phi ^2 _v + \phi ^2 _{\delta } + 2 \rho V_t \phi _x \phi _v ] C + \frac{1}{2} [ \phi ^2 _x + \phi ^2 _v + \phi ^2 _{\delta } + 2 \rho V_t \phi _x \phi _v ] C^2 =0. \end{aligned}$$

We obtain $C(t,T)=1$ and

$$\begin{aligned} {\left\{ \begin{array}{ll} \frac{\partial A}{\partial t} + \epsilon k_{\lambda } \theta _{\lambda } B + k_{\lambda } \theta _{\lambda } \kappa _1 (\xi ) + \kappa _2 (\xi ) =0 \\ \epsilon \frac{\partial B}{\partial t} - \kappa _1 (\xi ) k_{\lambda } - \epsilon k_{\lambda } B + \int _{-\infty }^{+\infty } [ e^{ \kappa _1 (\xi ) \beta + \epsilon B \beta + \xi z } -1 ] d\nu (z) =0. \end{array}\right. } \end{aligned}$$

Finally, by using conditions 6, we get:

$$\begin{aligned} {\left\{ \begin{array}{ll} \frac{\partial A}{\partial t} = - \epsilon k_{\lambda } \theta _{\lambda } B \\ \epsilon \frac{\partial B}{\partial t} = k_{\lambda } \epsilon B - e^{ \epsilon \beta B} +1. \end{array}\right. } \end{aligned}$$

We can repeat the same computation of the conditional moment generating function of $\lambda$ under ${\mathbb {P}}$ without the introduction of the terms $m_t$ and $m_T$ and we get the following system of ordinary differential equations:

$$\begin{aligned} {\left\{ \begin{array}{ll} \frac{\partial A}{\partial t} = - k_{\lambda } \theta _{\lambda } B \\ \frac{\partial B}{\partial t} = k_{\lambda } B - e^{\beta B} +1. \end{array}\right. } \end{aligned}$$

By comparing the equations written under ${\mathbb {Q}}$ and ${\mathbb {P}}$, the result follows. We can perform the computations of the conditional moment generating functions for all the remaining state variables $X_t, V_t, \delta _t$ (or alternatively of the joint conditional moment generating function), by following the procedure outlined above for $\lambda _t$, both under ${\mathbb {Q}}$ and ${\mathbb {P}}$, and, by direct comparison of the corresponding equations, the relations among the parameters under ${\mathbb {Q}}$ and ${\mathbb {P}}$ can be obtained. We omit all these computations, which are tedious and straightforward. In Appendix D an explicit solution is computed for the joint conditional moment generating function under ${\mathbb {Q}}$.

## Appendix D. Proof of Proposition 4

We define the joint moment generating function as

$$ \Psi ({\bar{u}},X_t, V_t, \delta _t, \lambda _t, t ,T)=\textrm{E}[e^{u_1 X_T + u_2 V_T + u_3 \delta _T + u_4 \lambda _T}|{\mathcal {F}}_t],$$

where ${\bar{u}}=(u_1, u_2, u_3, u_4)$. For $\tau =T-t$, by the Feynman-Kac theorem we get

D.1 $$\begin{aligned} \begin{aligned}&-\Psi _{\tau } + \left( - \frac{1}{2}V_t -\lambda _t \mu ^{\star } -\delta _t-\alpha X_t\right) \Psi _x + \frac{1}{2}V_t \Psi _{x x} + k_v(\theta _v-V_t)\Psi _v +\\&\quad +\frac{1}{2}\sigma ^2 V_t \Psi _{v v} + \rho \sigma V_t \Psi _{x v}+ k_{\delta }(\theta _{\delta }-\delta _t)\Psi _{\delta } + \frac{1}{2}\sigma _{\delta }^2 \Psi _{\delta \delta }+k_{\lambda }(\theta _{\lambda }-\delta _t)\Psi _{\lambda }\\&\quad +\lambda _t \int \left[ \Psi (u, X_t + J_x, V_t, \delta _t, \lambda _t+\beta , \tau ) - \Psi (u, X_t, V_t, \delta _t, \lambda _t, \tau )\right] \nu (dJ_x) =0. \end{aligned} \end{aligned}$$

Since the model is affine we can guess a solution of the form

$$\begin{aligned} \Psi ({\bar{u}},X_t, V_t, \delta _t, \lambda _t, \tau )&= \exp \Big ((u_1 + G(u_1, \tau ))X_t + A(u_1, u_2, u_3, u_4,\tau ) + \\&\quad + B(u_1, u_2,\tau )V_t + C(u_1, u_3,\tau )\delta _t + D(u_1, u_4, \tau )\lambda _t\Big ). \end{aligned}$$

For the jump transform we guess

$$\begin{aligned}{} & {} \Psi ({\bar{u}}, X_t + J_x, V_t, \delta _t, \lambda _t+\beta , \tau ) - \Psi ({\bar{u}}, X_t, V_t, \delta _t, \lambda _t, \tau )\\{} & {} \quad = \Psi ({\bar{u}}, X_t, V_t, \delta _t, \lambda _t, \tau )\left[ e^{u_1 J_x + \beta D(u_1,u_4,\tau )}-1\right] . \end{aligned}$$

Now, we need the partial derivatives of $\Psi$:

$$\begin{aligned} \Psi _{\tau }&=\Psi (A_{\tau }(u_1, u_2, u_3, u_4,\tau )+ B_{\tau }(u_1, u_2,\tau )V_t + C_{\tau }(u_1, u_3,\tau )\delta _t\\&\quad +D_{\tau }(u_1, u_4, \tau )+G_{\tau }(u_1, \tau )X_t), \\ \Psi _x&= \Psi (u_1+G(u_1, \tau )), \quad \Psi _v = \Psi B(u_1, u_2,\tau ), \\ \Psi _{vv}&= \Psi B(u_1, u_2,\tau )^2, \\ \Psi _{xv}&= \Psi \left[ B(u_1, u_2,\tau )(u_1+G(u_1, \tau )) \right] , \quad \Psi _{xx} = \Psi (u_1+ G(u_1, \tau ))^2,\\ \Psi _{\delta }&= \Psi C(u_1, u_3,\tau ),\\ \Psi _{\delta \delta }&= \Psi C(u_1, u_3,\tau )^2, \quad \Psi _{\lambda } = \Psi D(u_1, u_4,\tau ). \end{aligned}$$

By substituting the partial derivatives into (D.1) we obtain the following expression

$$\begin{aligned} \begin{aligned}&-\left( A_{\tau }(u_1, u_2, u_3, u_4,\tau )+ B_{\tau }(u_1, u_2,\tau )V_t + C_{\tau }(u_1, u_3,\tau )\delta _t\right. \\&\quad \left. +D_{\tau }(u_1, u_4, \tau )+G_{\tau }(u_1, \tau )X_t\right) \\&\quad + \left( - 0.5 V_t -\lambda _t \mu ^{\star } -\delta _t-\alpha X_t\right) (u_1+G(u_1, \tau )) \\&\quad + \frac{1}{2}V_t (u_1+ G(u_1, \tau ))^2 + k_v(\theta _v-V_t)B(u_1, u_2,\tau ) \\&\quad +\frac{1}{2}\sigma ^2 V_t B(u_1, u_2,\tau )^2 + \rho \sigma V_t \left[ B(u_1, u_2,\tau )(u_1+G(u_1, \tau )) \right] + k_{\delta }(\theta _{\delta }-\delta _t)C(u_1, u_3,\tau )\\&\quad + \frac{1}{2}\sigma _{\delta }^2 C(u_1, u_3,\tau )^2+k_{\lambda }(\theta _{\lambda }-\delta _t)D(u_1, u_4,\tau )\\&\quad +\lambda _t \int \left[ e^{u_1 J_x + \beta D(u_1,u_4,\tau )}-1\right] \nu (dJ_x) =0. \end{aligned} \end{aligned}$$

By separating the state variables we arrive at the ODE system of the thesis.

## Appendix E. Proof of Proposition 6

By the law of iterated expectations we get

$$\begin{aligned} m:=\textrm{E}[e^{u(X_{t_1} + \cdots + X_{t_n})}|{\mathcal {F}}_{t_0}] = \textrm{E}[e^{u(X_{t_1} + \cdots + X_{t_{n-1}})} \textrm{E}[e^{uX_{t_n}}|{\mathcal {F}}_{t_{n-1}}]|{\mathcal {F}}_{t_0}], \end{aligned}$$

then, from (11), replacing $u_1 = u$, $u_2=u_3=u_4=0$ and $\tau = t_{n} - t_{n-1}$, we get

$$\begin{aligned} m&= e^{A(u, 0, 0, 0,t_{n} - t_{n-1})}\textrm{E}\Big [e^{u(X_{t_1} + \cdots + X_{t_{n-1}})} \exp \Big ((u + G(u, t_{n} - t_{n-1}))X_{t_{n-1}} + \\&\quad + B(u, 0,t_{n} - t_{n-1})V_{t_{n-1}} + C(u, 0,t_{n} - t_{n-1})\delta _{t_{n-1}} + D(u, 0, t_{n} - t_{n-1})\lambda _{t_{n-1}}\Big )\Big |{\mathcal {F}}_{t_0}\Big ]. \end{aligned}$$

Applying again the law of iterated expectations

$$\begin{aligned} m&= e^{A(u, 0, 0, 0,t_{n} - t_{n-1})}\textrm{E}\Big [e^{u(X_{t_1} + \cdots + X_{t_{n-2}})} \\&\quad \textrm{E}\Big [\exp \Big ((2u + G(u, t_{n} - t_{n-1}))X_{t_{n-1}} + + B(u, 0,t_{n} - t_{n-1})V_{t_{n-1}} + C(u, 0,t_{n} - t_{n-1})\delta _{t_{n-1}} \\&\quad + D(u, 0, t_{n} - t_{n-1})\lambda _{t_{n-1}}\Big )\Big |{\mathcal {F}}_{t_{n-2}}\Big ] \Big |{\mathcal {F}}_{t_0}\Big ] \end{aligned}$$

we can solve the inner expectation using again (11), replacing $u_1 = (2u + G(u, t_{n} - t_{n-1}))$, $u_2= B(u, 0,t_{n} - t_{n-1})$, $u_3= C(u, 0,t_{n} - t_{n-1})$, $u_4=D(u, 0, t_{n} - t_{n-1})$ and $\tau = t_{n-1} - t_{n-2}$. Repeating these steps allows to solve all the expectations obtaining the recursive formulas of the thesis.

## Footnotes

1 We use the built-in Matlab^®^ function ncx2rnd.

2 We refer to Kyriakou et al. (Forthcoming) for more details on the algorithm, including practical implementation, runtime-accuracy studies and numerical performances (on several models) against competing methods.

3 Source: Refinitiv Eikon (formerly, Thomson-Reuters’ Datastream).

4 Data are taken from Barchart, derivatives on crude oil WTI are traded on the New York Mercantile Exchange (NYMEX), while derivatives on precious metals are traded on its commodity division (COMEX).

5 We use the built in Matlab^®^ function ode45.
